# An interchangeable prion-like domain is required for Ty1 retrotransposition

**DOI:** 10.1073/pnas.2303358120

**Published:** 2023-07-17

**Authors:** Sean L. Beckwith, Emily J. Nomberg, Abigail C. Newman, Jeannette V. Taylor, Ricardo C. Guerrero-Ferreira, David J. Garfinkel

**Affiliations:** ^a^Department of Biochemistry and Molecular Biology, University of Georgia, Athens, GA 30602; ^b^Robert P. Apkarian Integrated Electron Microscopy Core at Emory University, Atlanta, GA 30322

**Keywords:** retrotransposon, virus-like particle, prion-like domain, *Saccharomyces cerevisiae*

## Abstract

Retrotransposons and retroviruses shape genome evolution and can negatively impact genome function. *Saccharomyces cerevisiae* and its close relatives harbor several families of LTR-retrotransposons, the most abundant being Ty1 in several laboratory strains. The cytosolic foci that nucleate Ty1 virus-like particle (VLP) assembly are not well understood. These foci, termed retrosomes or T-bodies, contain Ty1 Gag and likely Gag-Pol and the Ty1 mRNA destined for reverse transcription. Here, we report an intrinsically disordered N-terminal prion-like domain (PrLD) within Gag that is required for transposition. This domain contains amino acid composition similar to known yeast prions and is sufficient to nucleate prionogenesis in an established cell-based prion reporter system. Deleting the Ty1 PrLD results in dramatic VLP assembly and retrotransposition defects but does not affect Gag protein level. Ty1 Gag chimeras in which the PrLD is replaced with other sequences, including yeast and mammalian prionogenic domains, display a range of retrotransposition phenotypes from wild type to null. We examine these chimeras throughout the Ty1 replication cycle and find that some support retrosome formation, VLP assembly, and retrotransposition, including the yeast Sup35 prion and the mouse PrP prion. Our interchangeable Ty1 system provides a useful, genetically tractable in vivo platform for studying PrLDs, complete with a suite of robust and sensitive assays. Our work also invites study into the prevalence of PrLDs in additional mobile elements.

## Significance

Retrovirus-like retrotransposons help shape the genome evolution of their hosts and replicate within cytoplasmic particles. How their building blocks associate and assemble within the cell is poorly understood. Here, we report a prion-like domain (PrLD) in the budding yeast retrotransposon Ty1 Gag protein that builds virus-like particles. The PrLD has similar sequence properties to prions and disordered protein domains that can drive the formation of assemblies that range from liquid to solid. We demonstrate that the Ty1 PrLD can function as a prion and that certain prion sequences can replace the PrLD and support Ty1 transposition. This interchangeable system is a useful platform to study disordered sequences in living cells.

Retrotransposons are pervasive across diverse eukaryotes and influence genome evolution and affect host fitness. The budding yeast *Saccharomyces cerevisiae* contains Ty1-5 long terminal repeat (LTR)-retrotransposons, with Ty1 as the most abundant element in many laboratory strains (1, 2). LTR-retrotransposons are the evolutionary progenitors of retroviruses; Ty1 elements share many structural hallmarks with retroviral genomic RNA and undergo an analogous replication cycle but lack an extracellular phase. Ty1 is transcribed from LTR-to-LTR and contains two partially overlapping open reading frames (ORFs): *GAG* and *POL*. Ty1 RNA serves as a template for protein synthesis and reverse transcription. Translation of Ty1 *POL* requires a programmed +1 frameshift near the C terminus of *GAG*, resulting in a large Gag-Pol precursor (p199) (3). Like retroviral RNA, Ty1 RNA is specifically packaged into virus-like particles (VLPs) where RNA is present in a dimeric form (3, 4). Proteolytic protein maturation occurs within VLPs by a protease (PR) encoded within *GAG* and *POL*. Ty1 PR cleaves the Gag-p49 precursor near the C terminus to generate p45, the capsid protein, and Gag-Pol-p199 to form mature PR, integrase (IN), and reverse transcriptase (RT) (3). Reverse transcription occurs within mature VLPs and, like HIV-1, requires a complex formed between RT and IN (3, 5). Ty1 preferentially integrates upstream of genes actively transcribed by RNA Polymerase III (Pol III) due to interactions between IN and Pol III subunits (3, 6–8).

Ty1 Gag performs the same functions as retroviral capsid and nucleocapsid. Amino acids 159 to 355 encode N-terminal domain (NTD) and C-terminal domain (CTD) capsid folds, assembling VLPs (9), and C-terminal sequences of Gag display nucleic acid chaperone (NAC) activity (10, 11). Sequences in the Ty1 RNA encoding the Gag protein are required for packing, dimerization, and reverse transcription (3). The N-terminal region of Gag has unknown function, and it is not known whether it is required for transposition.

While several steps of retrotransposon life cycles have been investigated, it is not well understood how their RNA genomes and protein machinery associate within the cellular milieu to facilitate VLP assembly and replication. Retroviral particle assembly often occurs in subcellular domains, referred to as “viral factories” or “viral inclusions” (12, 13). The sites of Ty1 VLP assembly are cytoplasmic foci termed retrosomes, or T-bodies, which contain Ty1 RNA, Gag, Gag-Pol, and perhaps additional cellular proteins (14–16). What drives the biogenesis of retrosomes is not understood. Mounting evidence suggests liquid–liquid phase separation (LLPS) underlies many examples of membraneless compartments (17, 18). Aggregation-prone proteins that drive LLPS have overlapping properties with prions, and both are implicated in age-related disease (19–26). Spontaneous demixing in these systems is often facilitated by intrinsically disordered domains, multivalent proteins, and scaffolding around nucleic acids. Indeed, prion-like and LLPS mechanisms provide intriguing models for retroelement assembly steps. Ty1 retrosomes contain Ty1 RNA and Gag oligomers associated with the RNA. Several viruses utilize LLPS in replication and assembly, including rabies virus (27), influenza A (28), herpes simplex virus 1 (29), measles virus (30), HIV-1 (31), and SARS-CoV-2 (32). Also, the human retrotransposon Long INterspersed Element-1 (LINE-1) has been reported to phase separate in vitro (33). Here, we present evidence that the Ty1 Gag protein contains a prion-like domain (PrLD) required for VLP assembly and transposition, raising the possibility that Ty1 Gag facilitates prion-like or phase-separating behaviors within retrosomes.

## Results

### Bioinformatic Analyses Reveal a PrLD in Ty1 Gag.

**Fig. 1.**
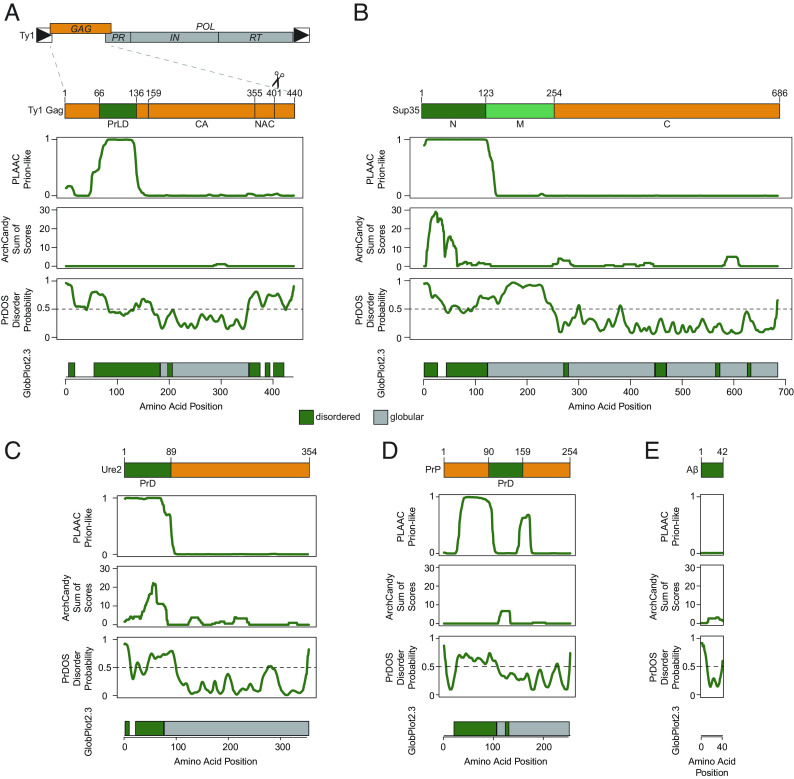
The Ty1 retrotransposon Gag contains a PrLD. Schematic of the Ty1 retrotransposon gene organization, with a detailed view of domains of the Gag protein (*A*), yeast prion Sup35 (*B*), yeast prion Ure2 (*C*), mouse prion protein (PrP) (*D*), and human amyloid beta (Aβ) (*E*); PrLD = prion-like domain, capsid domain (CA) and NAC domain are defined in ref. 9. Below are bioinformatic analyses of each protein aligned with the schematic above: yeast PLAAC, predicted amyloidogenic regions (ArchCandy), predicted protein disorder (PrDOS), predicted disordered (green) and globular (grey) regions (GlobPlot2.3).

Ty1 Gag contains several protein features, including capsid and NAC domains (9, 11). The N-terminal region of the protein, meanwhile, is predicted to be unstructured and does not have previously reported function. We analyzed Ty1 Gag (Fig. 1*A*) and Gag-Pol (*SI Appendix*, Fig. S1) using several bioinformatic tools designed to predict protein disorder, amyloidogenic secondary structures, and amino acid composition similarity to known yeast prions (34–37). For comparison, we ran the well-studied yeast prions Sup35 and Ure2, the mouse prion protein PrP, and Alzheimer’s disease–associated human Aβ_1-42_ through the same bioinformatic analyses (Fig. 1 *B*–*E*). Ty1 Gag contains a 71-amino acid domain with strikingly similar amino acid composition to yeast prions in its disordered N terminus, comparable to Sup35 and Ure2. This Gag PrLD is predicted to be unstructured by AlphaFold (38), and no published structures of the region are available, similar to canonical prions (9, 39–42) (*SI Appendix*, Fig. S2). Given the computational predictions and the requirement for Gag in forming Ty1 retrosomes, we further investigated prionogenic properties of the Gag PrLD, which we define as amino acid residues 66 to 136.

### Prionogenic Properties of the Gag_PrLD_.

**Fig. 2.**
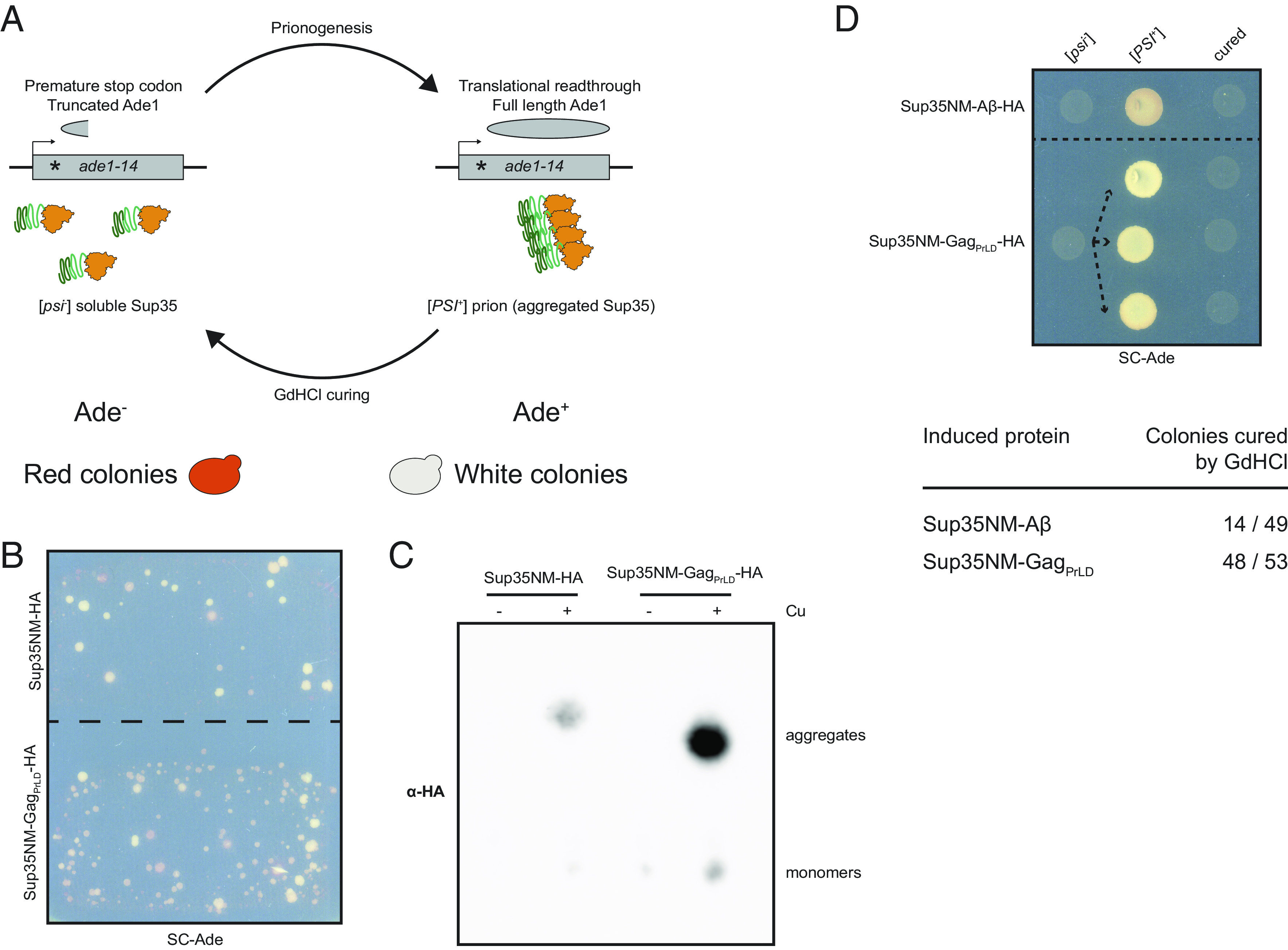
Gag_PrLD_ nucleates a Sup35-based prion reporter. (*A*) Schematic of the prionogenesis assay using the *ade1-14* allele containing a premature stop codon. Soluble Sup35 terminates translation at the premature stop codon, yielding a nonfunctional, truncated Ade1 (N-succinyl-5-aminoimidazole-4-carboxamide ribotide synthetase); yeast cannot grow on media lacking adenine (SC-Ade) and a red pigment develops. Sup35 aggregated into the prion state allows for translational read-through and production of functional Ade1; yeast grow on SC-Ade and appear white. (*B*) Qualitative prionogenesis of Sup35NM fusions; growth on SC-Ade indicates either a suppressor mutation or [*PSI^+^*] prionogenesis. Expression of Sup35 fusions were induced with 150 μM CuSO_4_. A representative image of at least three experiments is shown. (*C*) SDD-AGE analysis of Sup35NM-HA with and without Gag_PrLD_ fusion. Expression of Sup35 fusions were induced with 100 μM CuSO_4_. Monomers and high-molecular weight aggregates of chimeric proteins were detected with anti-HA antibody. A representative image of at least three experiments is shown. (*D*) Curing of Ade^+^ colonies by guanidine hydrochloride (GdHCl) of Sup35NM-HA chimeras. One [*psi^−^*] Sup35NM-Aβ fusion control strain is shown induced to [*PSI^+^*] and cured. Three independent inductions of a [*psi^−^*] Sup35NM-Gag_PrLD_ fusion are shown induced to [*PSI^+^*] and cured. [*PSI^+^*] yeast grow on SC-Ade while [*psi^−^*] and cured yeast do not. The table below shows the guanidine curability of Ade^+^ colonies induced by chimeric constructs.

We used a well-characterized Sup35-based in vivo reporter system to assess the ability of the Gag PrLD to promote prionogenesis in a yeast strain harboring a mutant allele of the adenine biosynthesis gene, *ade1-14*, which contains a premature stop codon (Fig. 2*A*) (43–45). Soluble Sup35 functions as a translation termination factor, resulting in a truncated nonfunctional Ade1 protein. Yeast fails to grow on media lacking adenine and appears red due to the buildup of a metabolic intermediate. However, formation of a prion state (termed [*PSI*^+^]) aggregates Sup35 away from the ribosome, allowing for translational read-through. This can be detected by adenine prototrophy and yeast colonies appearing white. Fusion of the PrLD of interest to the Sup35 N or NM domains promotes prion nucleation and has previously been used to study mammalian PrP and Aβ (45). Expression of Sup35NM-Gag_PrLD_ fusion under the *CUP1* copper-inducible promoter stimulates prionogenesis, as detected by increased papillation on SC-Ade when compared to the reporter alone (Fig. 2*B*). Protein expression and Ade^+^ growth is copper responsive; however, we found that the Sup35N reporter construct displays a high background growth upon induction (*SI Appendix*, Fig. S3 *A–E*). We next biochemically monitored prion aggregation using semidenaturing detergent–agarose gel electrophoresis (SDD-AGE) (46). Gag_PrLD_ fusions formed large, slow-migrating, copper-inducible aggregates with both Sup35N (*SI Appendix*, Fig. S2*F*) and Sup35NM (Fig. 2*C*) above reporter alone. Finally, we verified prion nucleation specifically, as opposed to colony growth due to accumulating suppressor mutations, by curing colonies of the prion after passaging cells on guanidine hydrochloride (GdHCl) (47, 48). Representative cells are shown for the naïve [*psi*^−^], induced [*PSI*^+^], and cured states for Sup35NM fusions to Gag_PrLD_ or positive control Aβ, both HA-tagged (Fig. 2*D*) and untagged (*SI Appendix*, Fig. S2*G*). A large fraction of Sup35NM-Gag_PrLD_ Ade^+^ colonies were curable by GdHCl.

### The Gag_PrLD_ Is Required for Ty1 Transposition.

**Fig. 3.**
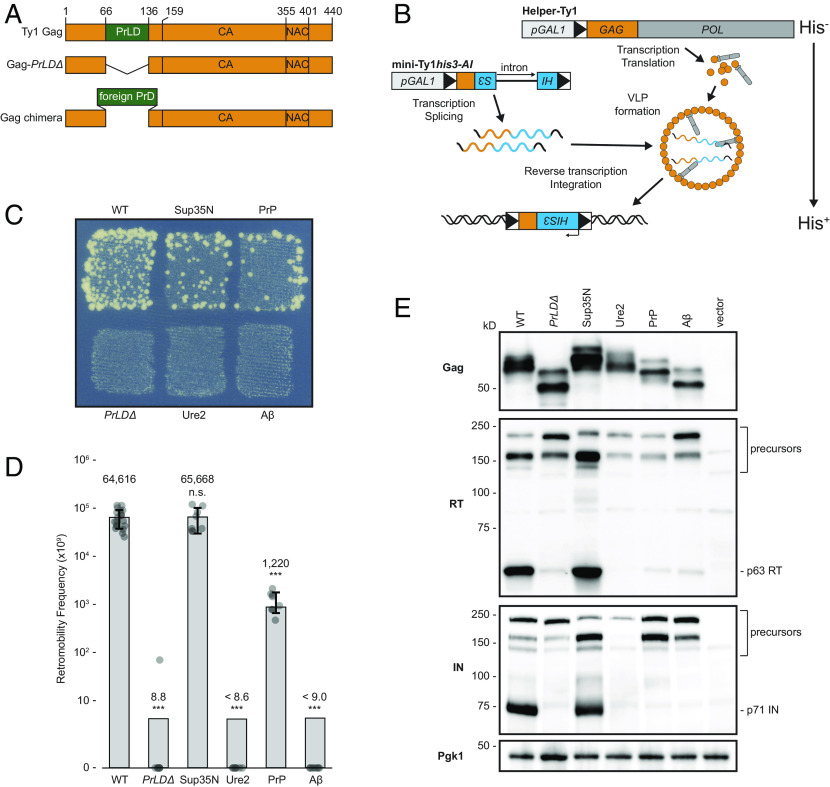
Ty1 Gag chimeras containing known PrDs produce stable Gag but have a range of transposition and proteolytic maturation phenotypes. (*A*) Schematic of Ty1 Gag constructs. The Ty1 Gag PrLD is intact in WT, deleted in *PrLDΔ*, and replaced with known PrDs in the chimeras. (*B*) Schematic illustrating the two-plasmid system separating Ty1 RNA and protein functions. Helper-Ty1 encodes a functional mRNA, providing protein products, but lacks a 3′ LTR thus disrupting *cis*-acting signals required for reverse transcription. Mini-Ty1*his3-AI* lacks complete ORFs but contains *cis*-acting signals for dimerization, packaging, and reverse transcription of mini-Ty1*his3-AI* RNA. The *his3-AI* indicator gene detects retromobility of mini-Ty1*HIS3* cDNA. (*C*) Qualitative retromobility of chimeric Gag constructs in the two-plasmid system. Colony growth on a medium lacking histidine indicates a retromobility event. A representative image of at least three replicates is shown. (*D*) Quantitative mobility assay of galactose-induced cells. Each bar represents the mean of at least eight independent measurements, displayed as points, and the error bar ± the SD. Error bars are omitted for *PrLDΔ*, Ure2, and Aβ chimeras that did not transpose; one retromobility event was observed in one replicate of *PrLDΔ*. Adjusted retromobility frequency is indicated above the bars. For Ure2 and Aβ, frequencies are indicated as less than the calculated frequency if one retromobility event had been observed. Significance is calculated from a two-sided Student’s *t* test compared with WT (n.s. not significant, ***P < 0.001. Exact *P*-values are provided in *SI Appendix*, Table S1). (*E*) Protein extracts prepared from galactose-induced cells expressing the indicated Gag constructs in the two-plasmid system were immunoblotted for the protein indicated on left. Polypeptide precursors are bracketed and mature RT and IN sizes are noted on right. Pgk1 serves as a loading control. Migration of molecular weight standards is shown alongside the immunoblots. A representative image of at least three replicates is shown.

Given that the Gag_PrLD_ promotes prionogenesis of a Sup35-based reporter, we investigated its functional role in Ty1 transposition. In a *Saccharomyces paradoxus* strain lacking genomic Ty1 elements (49, 50), we first deleted the PrLD from Gag in a Ty1 element provided on a plasmid and tagged with the robust and sensitive *his3-AI* retrotranscript indicator gene (51) (Fig. 3*A*). This marker contains a mutant *his3* gene split by an antisense artificial intron (AI) that is inserted at the 3′ untranslated region of Ty1 in the opposite transcriptional orientation. The AI is in the correct orientation to be spliced only in Ty1*his3-AI* RNA; cDNA reverse transcribed from this product results in a functional *HIS3* allele. Insertion into the genome, either by integration or recombination, allows cells to grow on media lacking histidine. The frequency of His^+^ prototrophy is a direct measure of Ty1*his3-AI* retrotransposition or cDNA recombination, collectively known as retromobility. Deletion of the Gag_PrLD_ in a complete Ty1*his3-AI* element overexpressed under the *GAL1* promoter completely abolished retromobility (*SI Appendix*, Fig. S4*A*), despite retaining similar Gag protein levels (*SI Appendix*, Fig. S4*B*). However, the PrLD region of Gag contains *cis*-acting RNA signals required for efficient reverse transcription (52, 53). To distinguish between a functional role in retrotransposition of the PrLD in the Gag protein versus the role of the RNA sequences that encode for the PrLD, we used a two-plasmid system to separate Ty1 RNA and protein functions (Fig. 3*B*). A helper-Ty1 encodes a functional mRNA, providing protein products, but lacks a 3′ LTR thus disrupting *cis*-acting signals required for reverse transcription. Mini-Ty1*his3-AI* lacks complete ORFs but contains *cis*-acting signals for dimerization, packaging, and reverse transcription of mini-Ty1*his3-AI* RNA (53, 54). Retromobility is monitored through the *his3-AI* reporter. In the two-plasmid assay, deletion of the Gag_PrLD_ also inhibits retromobility (Fig. 3 *C* and *D*), despite producing similar levels of Gag protein (Fig. 3*E*), confirming a critical contribution from the PrLD in the Gag protein to retromobility.

### Ty1 Mobility of Gag Chimeras Containing Foreign PrLDs.

To better understand the nature of the PrLD’s contribution to retromobility, we asked whether the Gag_PrLD_ sequence is uniquely capable of facilitating retromobility. Since the Gag_PrLD_ has prionogenic properties and sequence similarity to prions, we created chimeric Ty1 Gags in which the PrLD is replaced with prion domains from well-studied prions and aggregating proteins (Fig. 3*A*). We chose the yeast prions Sup35 and Ure2, the mouse prion protein PrP, and Alzheimer’s disease–associated human Aβ_1-42_ using domains predicted computationally (Fig. 1) (44, 45, 55). Chimeric Ty1 elements on the helper-Ty1 plasmid were coexpressed with mini-Ty1*his3-AI*, and the level of Ty1 mobility was determined. Remarkably, substitution of the Gag_PrLD_ with the prion domain from yeast Sup35 or mouse PrP supported Ty1 retromobility in qualitative (Fig. 3*C*) and quantitative retromobility assays (Fig. 3*D*). Gag_Sup35N_ retromobility is not significantly different from wild type (WT), whereas Gag_PrP_ is an order of magnitude lower, although still readily detectable on a qualitative plate assay. Replacing the PrLD sequence disrupts RNA signals, which is reflected in the single plasmid assay, in which Gag_Sup35N_ and Gag_PrP_ chimeras have dramatically reduced retromobility (*SI Appendix*, Fig. S4*A*), despite producing similar Gag protein levels (*SI Appendix*, Fig. S4*B*), highlighting the importance of separating protein and RNA function with the two-plasmid assay.

Retromobility measured as the frequency of His^+^ prototrophs formed from *his3-AI* tagged elements includes both new chromosomal integrations likely created via retrotransposition, and recombination of the spliced cDNA with homologous sequences present on the mini-Ty1*his3-AI* plasmid or solo LTRs present in the genome. To assess whether the chimeras support retrotransposition or merely recombination, we distinguished the two by, first, monitoring histidine prototrophy after segregating the helper and mini-Ty1*his3-AI* plasmids (*SI Appendix*, Fig. S4*C*). In our strain background with the WT two-plasmid system, 4% of retromobility events were due to recombination with either of the plasmids. The Gag_Sup35N_ and Gag_PrP_ chimeras had modestly increased recombination events, although only Gag_Sup35N_ reached statistical significance (*P* = 0.024) (*SI Appendix*, Fig. S4*D*). Secondly, we measured retromobility in a *rad52* mutant, thereby blocking Ty1 cDNA recombination (56, 57). *RAD52*-dependent recombination contributes to total retromobility of Gag_Sup35N_ and Gag_PrP_, but each also still support *RAD52*-independent retromobility (*SI Appendix*, Fig. S4*E*). Approximately one half of Gag_Sup35N_ retromobility events, and one-third of Gag_PrP_ events, are *RAD52*-independent. WT Gag had no statistically significant decrease in retromobility in a *rad52* mutant. We conclude that the Gag chimeras support de novo retrotransposition (56, 58).

### Effect of Gag_PrLD_ Chimeras on Ty1 Protein Level and Maturation.

The result that the Gag_PrLD_ can be replaced by foreign prion sequences indicates its function is not unique to the PrLD sequence and may be the same as provided in aggregation-prone proteins. However, not all the disordered domains tested in Gag chimeras supported transposition. Ty1 chimeras containing the domains from yeast Ure2 or human Aβ did not transpose (Fig. 3 *C* and *D*). All the chimeric Gags were expressed at similar levels (Fig. 3*E*), arguing against different transposition phenotypes due to effects on protein stability from the foreign prion domains. The substituted prion domains are of various sizes, and Gag chimeras had predicted electrophoretic mobilities. Gag proteolytically matures from p49 to p45 and is subject to posttranslational modifications, often resulting in multiple bands observed by western blot (3). To determine whether the Gag chimeras affected protein maturation, we assessed the relative levels of mature RT and IN by western blotting with antibodies specific to each protein. Deletion of the PrLD results in dramatically reduced mature RT and IN levels (Fig. 3*E*). The Gag_Sup35N_ chimera transposed as well as WT and produced equivalent levels of mature RT and IN. The transposition-deficient chimeras, Gag_Ure2_ and Gag_Aβ_, have very reduced levels, comparable to Gag*_PrLDΔ_*. Interestingly, Gag_PrP_ supports transposition, although reduced from WT, and has low levels of mature RT and IN. These results raise the possibility that Gag chimeras can block PR function and production of mature RT and IN that are essential for Ty1 mobility.

### Ty1 Gag_PrLDΔ_ and Gag Chimeras Fused to Green Fluorescent Protein (GFP) Affect Aggregation and Localization.

**Fig. 4.**
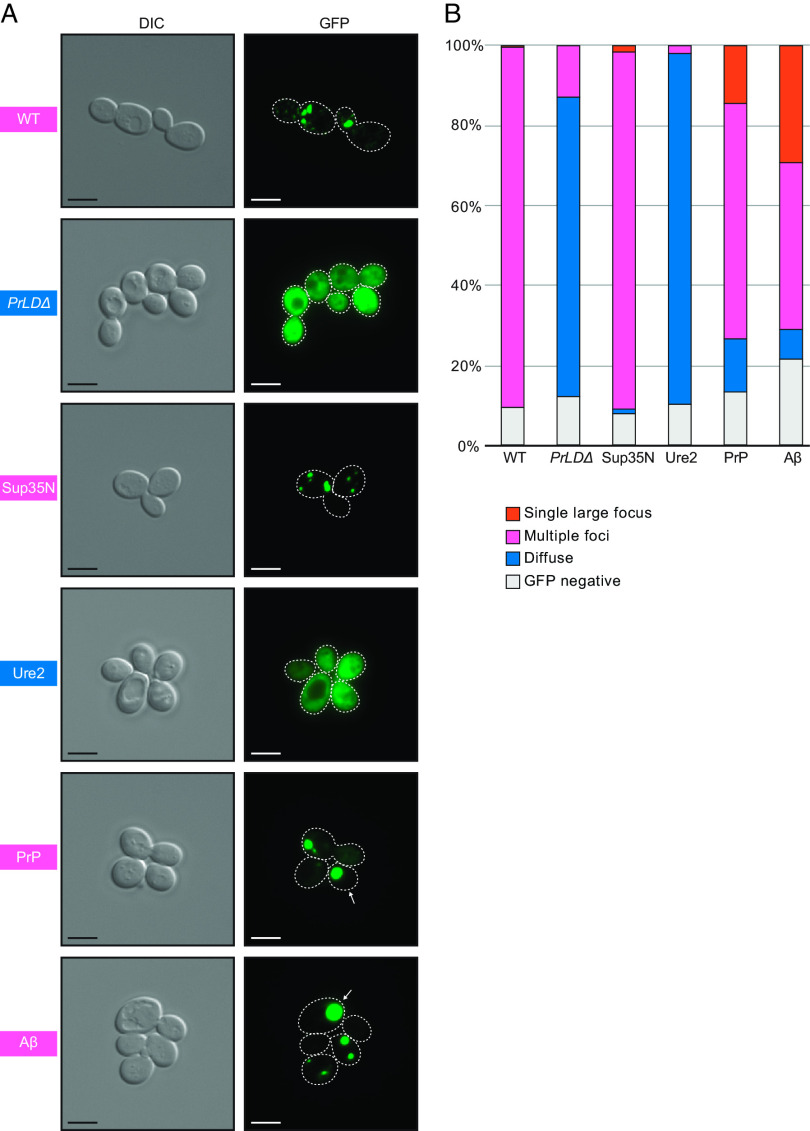
Foci detected in cells expressing WT Gag, the Gag*_PrLDΔ_*mutant, and Gag-PrLD chimeras fused to GFP. (*A*) Live-cell yeast fluorescence microscopy of strains expressing chimeric Gag-GFP after 24-h galactose induction. Differential interference contrast (DIC) and GFP channels are shown with cell outlines added to GFP channels based on DIC images. The strain labels are colored to match the most common foci observed. White arrows indicate cells with a single large focus. Scale bars represent 5 μm. (*B*) Quantitation of categories of foci observed as a percentage in at least 300 cells. The multiple foci category includes cells with multiple large foci, one or more small foci, or a combination of both sizes. Cell counts are provided in *SI Appendix*, Table S2.

Proteolytic maturation of RT and IN via PR occurs within VLPs (59), which are believed to be assembled in retrosomes (14–16). Gag fused to GFP has been used as a reporter for retrosome assembly and location (60), therefore we examined formation of cytoplasmic foci of WT, mutant, and chimeric Gag-GFP in the Ty-less background. WT Gag-GFP fusions formed discrete cytoplasmic foci, as previously reported using this construct, but deleting the PrLD resulted in diffuse localization throughout the cytoplasm (Fig. 4). We found that a 24-h galactose induction, shorter than 48-h induction used above, was ideal for live-cell microscopy and GFP-detection as yeast cultures are in log-phase growth (*SI Appendix*, Fig. S5). Twenty four-hour-induced Gag_Sup35N_ formed similarly discrete foci patterns as WT Gag, whereas Gag_Ure2_ had diffuse localization similar to Gag*_PrLDΔ_*. Gag_PrP_ supports transposition and predominately formed foci similar to WT, but also had a modest fraction of cells containing a visually distinct fluorescent morphology that appears as a single, large, very bright focus. Ty1 Gag_Aβ_ does not transpose, yet formed foci and an even larger fraction of cells contained these single, large foci. Forming Gag-GFP foci correlates with a requirement for transposition but, as Gag_Aβ_ shows, is not sufficient.

In addition, we investigated the structures formed by Gag-GFP chimeras in fixed yeast cells by thin-section transmission electron microscopy (TEM) (*SI Appendix*, Fig. S6) using methods similar to those used for detecting Ty1 VLPs (16). WT Gag-GFP produced electron-dense structures that appear similar to VLPs but look incomplete or incorrectly assembled, lacking a circular shell with a hollow interior. Gag_PrP_-GFP also produced clusters of structures reminiscent of VLPs, but in this case appearing denser and lacking a hollow interior. Gag*_PrLDΔ_*-GFP and Gag_Ure2_-GFP did not form any VLP-like structures detectable in micrographs. The Gag_Sup35N_-GFP strain produced tubular or filamentous structures, also not resembling proper VLPs. And strikingly, the Gag_Aβ_-GFP strain formed large densities in defined regions of the cell, instead of clusters of particles or filaments across the cytoplasm, perhaps corresponding to the single large foci seen by fluorescent microscopy. These results suggest that Gag-GFP can reveal severe assembly defects as evidenced by Gag*_PrLDΔ_*-GFP and Gag_Ure2_-GFP but GFP may confer aberrant VLP assembly properties when WT or chimeric Gag-GFP fusions are produced in cells.

### The Ty1 Gag_PrLDΔ_ and Gag Chimeras Affect VLP Assembly.

**Fig. 5.**
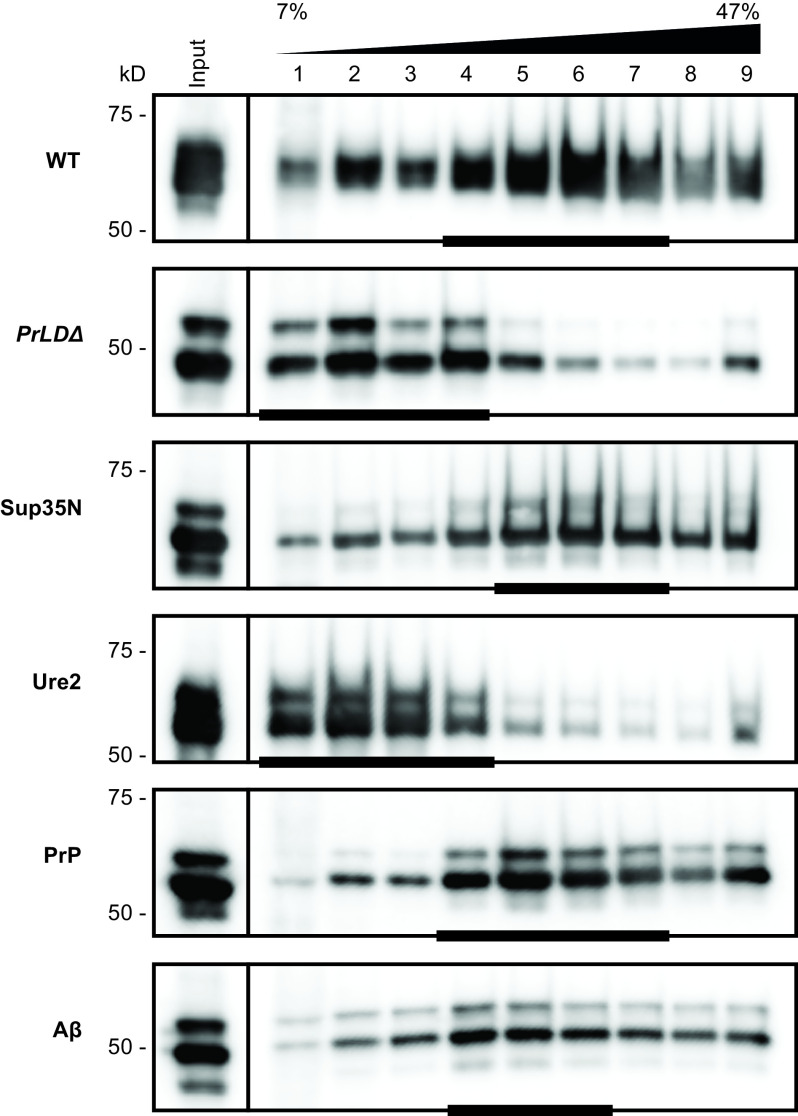
Transposition incompetent Gag chimeras disrupt VLP assembly. Protein extracts from galactose-induced yeast cells (Input) were fractionated over a 7 to 47% continuous sucrose gradient and immunoblotted for Gag. Expression plasmids and molecular weight standards are noted alongside the blots. The bars at the bottom of blots denote peak Gag fractions containing more than 1/9 of the Gag signal across the gradient, as determined by densitometric analysis. A representative image of at least three replicates is shown.

To evaluate VLP assembly in the chimeras using the two-plasmid system, we examined Gag sedimentation profiles of yeast lysate run through a 7 to 47% continuous sucrose gradient, as previously reported (9, 50, 61). WT VLPs accumulated in more dense sucrose fractions near the bottom half of the gradient, with peak fractions indicated by a bar (Fig. 5). Gag*_PrLDΔ_* appears unable to assemble complete VLPs, as Gag in these mutants accumulated in less dense sucrose fractions near the top of the gradient. The transposition-competent chimeras Gag_Sup35N_ and Gag_PrP_ had similar sedimentation profiles as WT, whereas transposition-deficient Gag_Ure2_ accumulated near the top of the gradient like Gag*_PrLDΔ_*. Gag_Aβ_ does not support retrotransposition, but peaked in similar fractions as WT, although somewhat more broadly distributed across the gradient.

**Fig. 6.**
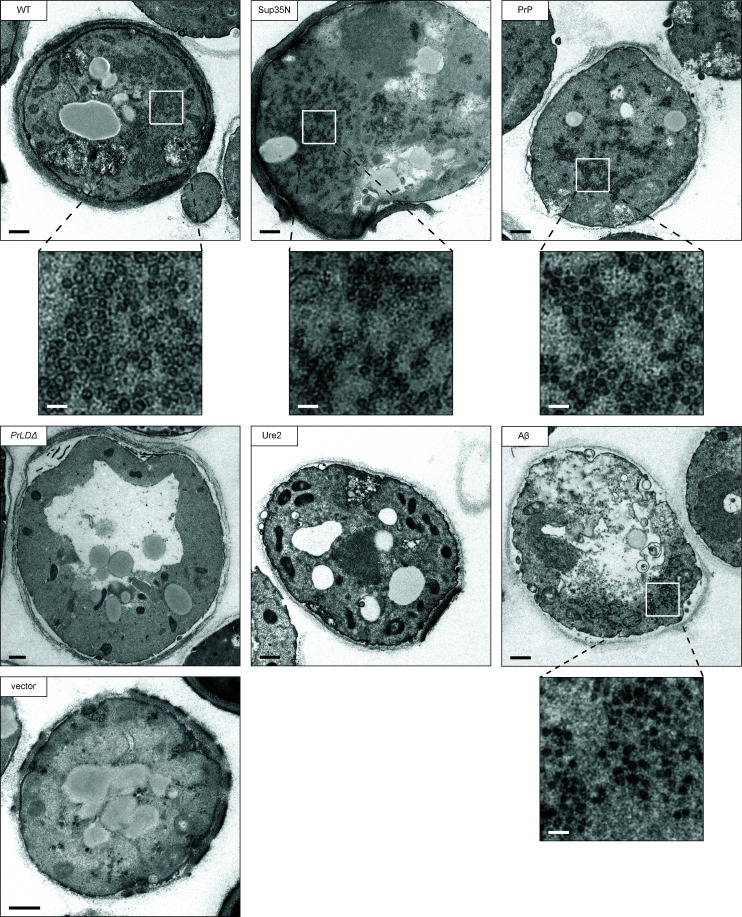
Transposition competent Gag chimeras support VLP production. Thin-section TEM of galactose-induced cells expressing Gag chimeras. Representative cells are shown, those containing VLP clusters include zoomed in cutouts to highlight VLPs. The black bars represent 500 nm, the white bars represent 100 nm.

To further examine the VLPs assembled by each chimera, we visualized thin sections of fixed yeast cells by TEM. Cells overexpressing the WT two-plasmid Ty1 system produced large clusters of VLPs (Fig. 6). VLPs are characteristically round with an electron-dense shell and their interior appears hollow in micrographs. Importantly, these particles were not observed in the parental yeast strain expressing empty vectors. Ty1 VLPs are heterogeneously sized and are approximately 30 to 80 nm in diameter, based on previous measurements of purified particles (62, 63). In thin-section TEM, particles may be in different Z-planes when sectioned, therefore masking the diameter of a roughly spherical particle, and preventing quantitative particle-size data collection from thin-section TEM. With this limitation in mind, we measured particle diameters from several cells in multiple micrographs to estimate an approximate size, and found WT particles ranging from 40 to 80 nm, with a median diameter of 59 nm (*SI Appendix*, Fig. S7), largely agreeing with previous reports of purified particles.

We did not observe any cells producing VLPs in the Gag*_PrLDΔ_* mutant, in agreement with Gag*_PrLDΔ_*-GFP imaging and sucrose sedimentation profiles. Taken together, these data lead us to conclude that the PrLD is required for Ty1 VLP assembly. The transposition-deficient Gag_Ure2_ chimera also did not assemble VLPs as monitored by thin-section TEM, again agreeing with sucrose sedimentation results. The two transposition-competent chimeras, Gag_Sup35N_ and Gag_PrP_, assembled VLPs similar in size and appearance to WT. These chimeras also produced large numbers of particles in each cell, although consistently appearing somewhat more dispersed throughout the cell than WT particle clusters. Interestingly, Gag_Aβ_ does not support retrotransposition, but has a similar sucrose sedimentation profile as WT, suggesting it may assemble particles that are defective for transposition. In thin-section TEM, we observed particles in cells expressing Gag_Aβ_ that are visually distinct from WT. The most striking difference is that these particles do not have the characteristic hollow center and instead appear electron-dense throughout. They are smaller than WT with a median diameter of 42 nm (*SI Appendix*, Fig. S7), and, like the Gag_Sup35N_ and Gag_PrP_ chimeras, are produced in large numbers of particles but are dispersed throughout the cell. Together, these results illustrate the robust and flexible nature of VLP assembly. However, our data also underscore the requirement for PrLD functionality as yeast and mammalian Gag-prionogenic chimeras form VLPs in vivo whereas the Gag*_PrLDΔ_*mutant does not.

## Discussion

The data presented here permit several conclusions about prionogenic domains, the functional organization of Ty1 Gag, and VLP assembly. Our results show that Ty1 Gag contains a PrLD that is required for VLP assembly and retrotransposition. The Gag_PrLD_ has intrinsic prionogenic properties as demonstrated by a cell-based Sup35 reporter, and its function in Ty1 transposition can be replaced by certain yeast and mammalian prion domains. Our findings also raise interesting questions about sequence constraints of PrLDs and how widespread PrLD functions are across retroelements. Furthermore, our work suggests Ty1 retromobility is an effective in vivo screening platform to study intrinsically disordered domains.

### Prion Properties of the Ty1 Gag_PrLD_.

We have examined prionogenic properties of the Ty1 Gag_PrLD_ using a cell-based assay in which Gag_PrLD_ is fused to the N and NM domains of Sup35 (45). Nonsense read-through is measured by auxotrophic growth and colony color, aggregate formation is monitored biochemically with SDD-AGE, and curability is assessed after GdHCl treatment. Further characterization of the Ty1 Gag_PrLD_ will include analyzing fusions to other Sup35 regions, measuring binding of the amyloid-sensitive dye thioflavin-T, and determining non-Mendelian inheritance (64, 65).

### RNA-Contributions to Gag_PrLD_ Function.

To determine protein-level effects of mutations in the Ty1 Gag PrLD from mutations of *cis*-acting RNA sequences, we separated RNA and protein function in a two-plasmid system (53, 54). Retromobility is lower in the two-plasmid system (Fig. 3*C*) than a single-plasmid expressing the intact transposon (*SI Appendix*, Fig. S4*A*). Gag_Sup35N_ restored retromobility in the two-plasmid system but had a severe retromobility defect in the single-plasmid assay. This is likely due to disruption of a functional pseudoknot in the RNA region that also encodes for the PrLD (52, 53).

### Sequence Requirements of the Ty1 Gag_PrLD_.

We replaced the Gag_PrLD_ with exogenous prion domains based on computational predictions and functional analyses. We chose the entirety of Aβ_1-42_ and the complete N-terminal domain of Sup35_2-123_. We introduced the highest scoring 60 amino acid stretch predicted by prion-like amino acid composition (PLAAC), Ure2_17-76_, which is within the established prion domain reported as the first 89 amino acids (44, 55). The infectious PrP 27-30 isoform contains about 142 amino acids and spans 90 to 230 (66), but shorter truncations still display prion phenotypes (67–69), and PrP_90-159_ is sufficient to induce prionogenesis in yeast (45). PrP_121-231_ is soluble, and its structure has been determined using solution NMR (41, 70). We introduced PrP_90-159_ as a Ty1 Gag chimera based on prior success in yeast. It will be interesting to examine other regions of PrP for function when present in Gag.

Although the sequence features constraining Ty1 PrLD function are not well defined, both the transposition-competent Gag chimeras (Sup35 and PrP) are from proteins with oligopeptide repeats associated with prionogenesis (71, 72). However, the PrP sequence introduced as a Ty1 Gag chimera does not contain these repeats. Moreover, the Ty1 Gag_PrLD_ does not have equivalent repeats of 8 to 10 amino acids. Instead, like other reported prion domains, the Gag_PrLD_ is Q/N-rich and is depleted of charged residues. Additionally, a large number of prolines in the Gag_PrLD_ likely prevents secondary structure formation and contrasts with the highly alpha-helical folding of the Gag capsid domain (9, 61). Further investigation will be required to understand the sequence parameters, such as length, amino acid composition, charge, or oligorepeats, that govern function of the Gag_PrLD_.

### Gag Chimeras Reveal Varied Properties across the Ty1 Life Cycle.

Importantly, the Gag_Sup35N_ chimera restored Ty1 mobility, produced VLPs with WT morphology and sedimentation profiles, as well as mature IN and RT. Gag_PrP_ supported retromobility, although less well than WT or Gag_Sup35N_. Whereas Gag_PrP_ produces VLPs with WT morphology by TEM (Fig. 6) and similar sedimentation profiles (Fig. 5), Gag_PrP_ accumulates low levels of mature RT and IN (Fig. 3*E*). This could indicate an incompatibility of Gag_PrP_ as a substrate for PR. Another possibility is that Gag_PrP_ VLPs inefficiently incorporate Gag-Pol or are partially defective in ways not detectable by TEM or sedimentation. The reduced retromobility of Gag_PrP_ may be explained by impaired RT and IN protein maturation. Meanwhile, Gag_Aβ_ produces morphologically altered particles that do not support retrotransposition or Pol maturation. These particles lack the characteristic hollow center observed in TEM of WT VLPs (Fig. 6) and are noticeably smaller in diameter (*SI Appendix*, Fig. S7). These observations highlight the robust nature of VLP assembly but reveal that simply assembling particles is not sufficient for transposition or protein maturation. It will be informative to measure packaging of the mini-Ty1 RNA into chimeric VLPs as productive reverse transcription requires both mature enzymes, a correctly folded RNA substrate, and the tRNA_Met_ primer to be present in VLPs. Our sedimentation and TEM results build upon previously published sedimentation experiments (9, 50, 61), and strengthen the value of sedimentation as a proxy for VLP assembly. Nonetheless, the value of TEM is exemplified by the Gag_Aβ_ chimera, which sediments similarly to WT but TEM reveals aberrant particle morphology.

Gag-GFP fusions are used as a proxy for Ty1 retrosomes (60), although we have not formally tested for Ty1 RNA colocalization in our system. We used a previously characterized WT GFP-fusion construct that contains the mature Gag (p45) and not a full-length element (60). Here, the utility of Gag-GFP is shown by the lack of foci for the Gag*_PrLDΔ_* mutant in growing cells and the cellular mislocalization observed in Gag chimeras (Fig. 4). However, GFP is a 26 kD protein and fusion impaired proper VLP formation (*SI Appendix*, Fig. S6), perhaps by interfering with Gag-Gag contacts required for assembly of a complete particle structure. Examining the PrLD fused to GFP alone, without the full Gag protein, or testing a Gag truncation that lacks the NAC domain, will indicate the minimal region that promotes foci formation and whether RNA recruitment is required. Ty1 Gag contains a NAC and binds Ty1 RNA, but also binds diverse RNAs in vitro and cellular mRNAs associate with Ty1 VLPs (10, 11, 54, 73–75). Whether Ty1 RNA, specifically, is required to form foci or to nucleate VLP assembly, or whether there is an RNA requirement at all, requires further study. Further studies are required to understand the kinetic components and cytoplasmic localization required to form retrosomes and their progression toward VLP assembly, but dynamic transitions are evident in our Gag-GFP analyses and retrograde transport of Gag from the endoplasmic reticulum is required for protein stability (60).

### Does the Ty1 Retrosome Constitute a Phase-Separated Compartment?

WT cells assemble discrete VLPs that can be found throughout the cell but are often observed in a particular region of the cytoplasm, and even the WT Gag-GFP-assembled discrete structures, observed by TEM. However, the Gag_Aβ_-GFP strain produced large densities that may correspond to large foci observed by fluorescence microscopy. These assemblies would be consistent with LLPS compartments containing high concentrations of Gag-GFP that stall and cannot complete VLP assembly; however, we have not examined LLPS properties such as concentration dependence, droplet merging, or internal mixing (17). PrLDs can drive formation of a gradient of assemblies, from LLPS to hydrogels and amyloid-like fibers. The Ty1 Gag chimeras may exhibit a spectrum of these morphologies. The filamentous assemblies formed by Gag_Sup35N_-GFP are potentially similar to Sup35 amyloid fibers observed in vitro, and Gag_Aβ_-GFP may form liquid droplets. Sup35, while canonically known for its ability to form amyloid fibers as a prion, has more recently been appreciated to undergo LLPS upon a decrease in cytosolic pH and can mature over time into a gel-like condensate (76, 77). Whereas WT Gag allows for VLP assembly to proceed and supports transposition, perhaps transiently existing in an LLPS state, chimeras may become blocked along the retrosome and VLP assembly pathway, resulting in the striking structures observed by fluorescence microscopy and TEM. Further work will be required for the rigorous characterization necessary to declare the Ty1 retrosome or other assemblies formed by Gag chimeras an LLPS compartment. Ty1 provides a promising system to unite studies of prion and LLPS pathways.

### An Interchangeable Platform to Study PrLD and LLPS Domains in Living Cells.

The condensate-forming property, but not the prion-forming property, of Sup35 is conserved across 400 My from *S. cerevisiae* to *Schizosaccharomyces pombe*, emphasizing the evolutionary importance of this ancient phenotype (76). Our finding of the Ty1 PrLD raises the possibility that LLPS may be widespread among retroelements. Our preliminary computational analyses of Pseudoviridae (Ty1/copia) retroelement family members reveal predicted PrLDs in not only the closely related yeast Ty2, but also in distantly related plants in the *Oryza* element *Retrofit* and the *Arabidopsis* elements *Evelknievel* and AtRE1. The human retrotransposon LINE-1 phase separates, and retrotransposition is associated with cancer (78) and age-associated inflammation (79, 80). A condensate-hardening drug was found to block human respiratory syncytial virus replication, which occurs in virus-induced inclusion bodies (81), highlighting the potential of the Ty1 platform to contribute to new antiviral and other human-health therapeutics. The Ty1 Gag chimera strategy developed here may prove to be a useful platform to study prion-like and LLPS-forming domains due to the genetic tractability of yeast, the presence of host modulators (3), and the suite of robust and sensitive in vivo assays developed for Ty1.

## Materials and Methods

### Bioinformatic Analyses.

Detailed descriptions of PLAAC, ArchCandy, PrDOS, GlobPlot2.3, and structure analyses are provided in *SI Appendix*, *Materials and Methods*.

### Yeast Strains and Plasmids.

Yeast strains with full genotypes are listed in *SI Appendix*, Table S3. Plasmids, primers, and gene fragments are listed in *SI Appendix*, Tables S4–S6. Detailed descriptions of strains, media, plasmids, and cloning are provided in *SI Appendix*, *Materials and Methods*.

### Prion Assays.

Prion nucleation, curing, and semidenaturing detergent–agarose gel electrophoresis were adapted from published methods (45, 46). Detailed protocols are described in *SI Appendix*, *Materials and Methods*.

### Ty1 Mobility, Protein, and Microscopy Analyses.

Ty1 retromobility events were detected using the *his3-AI* retromobility indicator gene (51) as previously described (9, 50). Total yeast protein was prepared by trichloroacetic acid precipitation and immunoblotted using standard techniques (50, 82). Sucrose gradient sedimentation was performed as previously described (9). Live-cell fluorescence microscopy and TEM were performed using standard techniques and adapted from published methods (16). Detailed protocols are described in *SI Appendix*, *Materials and Methods*.

## Supplementary Material

Appendix 01 (PDF)

## Data Availability

All study data are included in the article and/or *SI Appendix*.
