# Supplementary material for: An interchangeable prion-like domain is required for Ty1 retrotransposition
Source: Proc Natl Acad Sci U S A. 2023 Jul 17;120(30):e2303358120. doi: 10.1073/pnas.2303358120 (PMC10372613; doi:10.1073/pnas.2303358120)
Supplement: Supplementary file 1 — Appendix 01 (PDF) [file pnas.2303358120.sapp.pdf]

PNAS

Supporting Information for
An interchangeable prion-like domain is required for Ty1 retrotransposition

Sean L. Beckwith[1], Emily J. Nomberg[1], Abigail C. Newman[1], Jeannette V. Taylor[2], Ricardo C. Guerrero-Ferreira[2], and David J. Garfinkel[1]

[1]Department of Biochemistry and Molecular Biology, University of Georgia, Athens, GA, 30602, USA.
[2]Robert P. Apkarian Integrated Electron Microscopy Core at Emory University, Atlanta, GA, 30322, USA.

David J. Garfinkel
Email: djgarf@uga.edu

This PDF file includes:

Supporting text
Figures S1 to S7
Tables S1 to S6
SI References

SI Appendix

SI Materials and Methods

Fig S1. PrLD predictions for Ty1.

Fig S2. Prionogenic domains are intrinsically disordered in experimental and predicted protein structures.

Fig S3. Gag_{PrLD} nucleates a Sup35-based prion reporter.

Fig S4. Gag chimeras likely disrupt Ty1 RNA functions and increase cDNA recombination with plasmid-borne mini-Ty1*his3-AI*.

Fig S5. Chimeric Gag-GFP after 48 hr galactose induction.

Fig S6. Thin-section TEM of Gag-GFP strains.

Fig S7. VLP diameter of Gag-PrLD chimeras.

Supplementary Table S1. Retromobility frequencies.

Supplementary Table S2. Gag-GFP chimera fluorescent microscopy cell counts.

Supplementary Table S3. Yeast strains used in this study.

Supplementary Table S4. Plasmids used in this study.

Supplementary Table S5. Primers used in this study.

Supplementary Table S6. Gene fragments used in this study.

SI References

SI Materials and Methods

Bioinformatic analyses. PLAAC (http://plaac.wi.mit.edu/) (1) was used with default settings: core length of 60 and 100% *S. cerevisiae* background probabilities. ArchCandy (https://bioinfo.crbm.cnrs.fr/index.php?route=tools&tool=7) (2) was used with a score threshold of 0.500 and the transmembrane regions filter off; the sum of scores data is presented. PrDOS (https://prdos.hgc.jp/) (3) was used with the default 5% FDR and the disordered probability threshold set to 0.5. GlobPlot2.3 (http://globplot.embl.de/) (4) was used with default settings with Russell/Linding propensities. Bioinformatic outputs were uniformly plotted using a custom script using the base plot() and rect() functions in R version 3.5.2. Structure analysis was performed using PyMOL v1.5.0.5 with the "align" command.

Yeast strains and media. Standard yeast genetic and microbiological techniques were used in this work (5). Prion nucleation experiments were performed in GT409, an *S. cerevisiae* strain that is [*psi*$^-$ *pin*$^-$] and harbors the *ade1-14* allele which contains a premature stop codon (kindly provided by Y. Chernoff) (6). Ty1 assays were performed in the DG3582 background, a Ty-less *S. paradoxus* derivative of DG1768 (7, 8). Rad52-dependence was tested in the DG2204 background, a Ty-less spore derived from a cross between DG2196 and DG1768 (9). For galactose induction in liquid media, starter cultures were grown overnight at 30 °C in synthetic complete (SC) dropout media containing 2% raffinose, diluted 1:20 into media containing 2% galactose, and grown at 22 °C for 24-72 hours.

Plasmids and cloning. Plasmids, primers, and gene fragments are listed in Supplementary Tables 4-6. All Ty1 nucleotide and amino acid information correspond to the Ty1H3 sequence (GenBank M18706.1). All cloning was done with NEBuilder HiFi DNA Assembly Master Mix (New England Biosciences cat. no. E2621). Sup35 fusion plasmids pBDG1691 (1434), pSLBB027 (1134), and pSLBB028 (1258), driven by the *CUP1* promoter, were kindly provided by Y. Chernoff (Chernoff lab plasmid nomenclature in parentheses). Sup35N plasmids contain Sup35 amino acids 1-123, and Sup35NM contains amino acids 1-250. The Gag_{PrLD} contains Gag amino acids 66-136. Gag_{PrLD} fusions to Sup35 were subcloned via EcoRI and XbaI digest and PCR from pBDG598 using primers SLBP0045-7. Hemagglutinin epitope (HA) tags were inserted via XbaI and SacII digest using ssDNA oligos AB42-HA (SLBP0088) or GagPrLD-HA (SLBP0087) and HAtag-SacII (SLBP0086).

pBDG1647 was kindly provided by K. Pachulska-Wieczorek and is the mini-Ty1*his3*-AI plasmid (pJC994) which was constructed by deleting the HpaI-SnaBI fragment of pGTy1*his3AI-[Δ1]* (nucleotides 818-5463 of Ty1-H3) (10).

pBDG1781 contains pGTy1nt.241-5561 which is pEIB ("enzyme-in-a-box"). pEIB was kindly provided by J. Strathern. It was created by deleting the BglII-NcoI fragment which removes the U3 polypurine tract (PPT) and 3' LTR, preventing reverse transcription of the Ty1 RNA produced from pEIB. The original pEIB provided by J. Strathern also contained a multiply mutated primer binding sequence (PBS), disrupting complementarity to the intitiator $tRNA_i^{Met}$ which primes reverse transcription. pBDG1781 was corrected back to the original Ty1H3 PBS sequence via XhoI and HpaI digest and PCR from pBDG598 using primers SLBP0116-7.

pBDG1781 derivatives were generated by replacing the Gag_{PrLD} with custom commercial gene fragments (Integrated DNA Technologies (IDT) and Twist Bioscience) via XhoI and HpaI digest. *PrLDΔ* was cloned using SLBG0030 and the chimeras were cloned using overlapping gene fragments SLBG0024, SLBG0025 and a gene fragment encoding the foreign prion domain. The $A\beta_{1\text{-}42}$ sequence used is identical to that in pBDG1691 provided by Y. Chernoff and contains a silent mutation at codon 3 (GAA>GAG) to remove an EcoRI site. Mouse PrP (UniProt P04925) amino acid sequence was codon optimized for S. *cerevisiae* using the IDT codon optimization tool.

pBDG1799 contains mature Gag (amino acids 1-401) driven by the *GAL1* promoter fused to GFP-(S65T) with a 7 amino acid linker (nt. CGGATCCCCGGGTTAATTAAC) followed by the *ADH1* terminator sequence, which was kindly provided by J. Curcio on plasmid BJC1066, which is in a pRS415 backbone. The expression construct was subcloned to pRS413 (11) using primers SLBP0221-2 and inserted via XhoI and SacII digest to create pBDG1799. Derivatives were subcloned via XhoI and BbvCI digest and PCR from the corresponding chimeric pEIB plasmids using primers SLBP0117 and SLBP0194.

pBDG598 is pGTy1mhis3-AI, described in (12), and is driven by the *GAL1* promoter and is marked with the *his3*-AI retrotranscript indicator gene. Derivatives were subcloned via XhoI and HpaI digest and PCR from the corresponding chimeric pEIB plasmids using primers SLBP0116-7. All plasmids generated were verified by DNA sequencing.

Prion nucleation and curing. [*PSI*+] induction was assayed in a [*psi*-] strain for chimeric plasmids under a P_{CUP1} promoter; yeast cells were grown at 30 °C. Yeast were grown on SC-Ura for 2 days, replica plated to SC-Ura ± 150 μM $CuSO_4$ and grown for 2 days, then replica plated to SC-Ade and grown for approximately 10 days until imaged. Following prion nucleation, Ade^+ colonies were cured of [PSI^+] by guanidine hydrochloride (GdHCl). First, the induction plasmid was counter selected on FOA and single colonies were isolated. Then, Ade^+/Ura^- colonies were passaged as single colonies on YPD spotted with 10 or 25 μL of 5 M GdHCl until red-pigmented colonies developed.

SDD-AGE. Semi-denaturing detergent-agarose gel electrophoresis (SDD-AGE) was adapted from published methods (6, 13). Yeast were subcultured from an overnight SC-Ura starter culture into SC-Ura ± 100 μM $CuSO_4$ and grown overnight at 30 °C. Approximately 1 x 10^8 cells were lysed in 200 μL of ice cold Buffer A (50 mM Hepes, pH 7.5; 150 mM NaCl; 2.5 mM EDTA; 1% Triton X-100) with 400 μg/mL PMSF, 16 μg/mL each of aprotinin, leupeptin, pepstatin, and 6 mM DTT by vortexing with glass beads twice for 5 minutes at 4 °C. Cell debris was removed by centrifugation for 2 minutes at 5000 rpm at 4 °C. 4X sample buffer (2X TAE; 20% glycerol; 4% SDS; bromophenol blue) was added to the supernatant and run on a 13 cm 1.8% agarose gel containing 1X TAE and 0.1% SDS at 50 V for several hours until the dye front reached 1 cm from the bottom of the gel. Proteins were transferred to PVDF using 1X TBS by downward capillary transfer overnight at room temperature. The membrane was immunoblotted by standard immunoblotting.

Ty1*his3-AI* mobility assays. Ty1 retromobility events were detected using the *his3-AI* retromobility indicator gene (12) by qualitative and quantitative assays (8). Qualitative assays

were printed from glucose plates onto galactose plates, grown for 48 h at 22 °C, then printed to glucose plates lacking histidine and grown at 30 °C. Quantitative retromobility frequencies were determined from galactose inductions diluted in water, plated on synthetic dropout media, and colonies counted. All experiments were galactose-induced for 48 h at 22 °C, except SLBY460-5 were induced for 72 h. Data represent at least 4 independent galactose inductions (replicate counts provided in Supplementary Table S1); *p*-values were calculated by two-sided Student's *t*-test. Determination of likely cDNA recombinants versus likely genomic insertions by segregation test was conducted on His^+ papillae isolated after 48 hr galactose induction. The *URA3*-bearing plasmid was counter-selected by growth on media containing 5-fluoroorotic acid. Cells that had lost the *TRP1*-bearing plasmid after single colony passaging on YPD were determined by printing to SC-Trp plates. Ura^-/Trp^- cells were tested for growth on SC-His. Loss of the His^+ phenotype concomitant with plasmid loss indicates a likely cDNA recombinant since the only complete Ty1 sequence present for homologous recombination is on the plasmids. Retention of the His^+ phenotype indicates a likely genomic insertion. *p*-values were calculated by Fisher's exact test compared to wildtype. 100 retromobility events was selected for feasibility of data collection after estimating required sample size of 126 by *a priori* power analysis to detect increased recombination frequency of a 10% effect size with 80% power compared with a 5% recombinant frequency in wildtype piloted with 20 retromobility events. Power analysis for Fisher's exact test was performed using G*Power 3.1 (14).

Immunoblotting. Total yeast protein was prepared by trichloroacetic acid (TCA) precipitation using standard techniques (8, 15). Briefly, cells were broken by vortexing in the presence of glass beads in 20% TCA and washed in 5% TCA. Proteins were separated on 8% or 10% SDS-PAGE gels. PVDF membranes were immunoblotted with antibodies at the following dilutions in 2.5% milk-TBST: mouse monoclonal anti-HA antibody clone 2-2.2.14 (Invitrogen cat. no. 26183) (1:1000), mouse monoclonal anti-TY tag antibody clone BB2 (kindly provided by S. Hajduk) (1:10,000) (16), mouse monoclonal anti-IN clone 8B11 (kindly provided by J. Boeke) (1:1,000), rabbit polyclonal anti-RT (Boster Bio cat. no. DZ33991) (1:500), or mouse monoclonal anti-Pgk1 antibody clone 22C5D8 (Invitrogen cat. no. 459250) (1:1000). Immune complexes were detected with WesternBright enhanced chemiluminescence (ECL) detection reagent (Advansta cat. no. K-12049-D50). All imaging was done using a ChemiDoc MP (Bio-Rad). Precision Plus Kaleidoscope protein standards (Bio-Rad cat. no. 1610395) were used to estimate molecular weights.

Live cell fluorescence microscopy. Following 24 or 48 hr galactose induction, cells were imaged directly in growth media on positively charged slides (Globe Scientific cat. no. 1358W) using a Zeiss Axio Observer.Z1 epifluorescence microscope equipped with an AxioCam HSm camera and captured using AxioVision v4.8.2 software (Carl Zeiss Microscopy).

Sucrose gradient sedimentation. Following 48 hr galactose induction, a 100 mL culture was harvested, and cells were broken in 15 mM KCl, 10 mM HEPES- KOH, pH 7, 5 mM EDTA containing RNase inhibitor (100 U/mL), and protease inhibitors (16 µg/mL aprotinin, leupeptin, pepstatin A and 2 mM PMSF) in the presence of glass beads. Cell debris was removed by centrifuging the broken cells at 10,000 x g for 10 min at 4°C. Clarified whole cell extract in 500

μL of buffer was applied to a 7-47% continuous sucrose gradient and centrifuged using an SW41 Ti rotor at 25,000 rpm (77,000 x g) for 3 hr at 4°C. After centrifugation, 9 x 1.2 mL fractions were collected, and input and fractions were immunoblotted with TY-tag antibody to detect Gag. Densitometric analysis was performed using Image Lab (Bio-Rad, v. 6.0.1).

Transmission electron microscopy preparation and imaging of yeast cells. Following 48 hr galactose induction, or 24 hr induction for GFP-expressing strains, cells were fixed with 4% formaldehyde - 2.5% glutaraldehyde in 0.1 M sodium cacodylate pH 7.4 for 2 hr at 4 °C, washed three times with cold PBS, once with cold 0.1 M KPO_4 (pH 6.5), and once with cold P solution (1.2 M sorbitol, 0.1 M KPO_4 pH 6.5). Cells were spheroplasted in P solution with 25 mM DTT for 15 min at 37 °C using 400 μg/mL of Zymolyase-20T. Spheroplasts were gently washed three times with cold PBS, stored in 0.1 M sodium cacodylate pH 7.4 at 4 °C, and transported to the Robert P. Apkarian Integrated Electron Microscopy Core. Then the cells were washed in fresh 0.1 M cacodylate buffer and spun for 5 minutes at 8000 rpm on an Eppendorf Centrifuge 5430. The cells were spun between each step and were processed in the microcentrifuge tubes in which they were received. After two 0.1 M cacodylate buffer washes of ten minutes each, the cells were post-fixed for an hour in 1% buffered osmium tetroxide. Following two ten-minute washes in distilled water, the cells were en-bloc stained with 0.5% Uranyl Acetate in 0.1 M sodium acetate for 30 minutes. The cells were washed in distilled water for 10 minutes, and then dehydrated in an ascending ethanol series of 15-minute steps starting with 25% and ending with 100% ethanol followed by two 15-minute steps of propylene oxide (PPO). The cells were infiltrated with Eponate12 (Ted Pella, Inc.) epoxy resin in four steps: 1:2 of resin to PPO, 1:1 resin to PPO, 2:1 resin to PPO, and two changes of 100% resin. All resin steps were for 4 hours to overnight, followed by a final change of fresh 100% resin. The cells in resin were then polymerized for two to three days at 60 °C. After release from the tubes, the sample blocks were faced. Ultrathin sections of 70 to 80 nm were made using a Reichert Ultracut S and a Diatome diamond knife. The sections were collected onto 200 mesh copper grids with Carbon stabilized Formvar™ support film then post-stained with 5% Uranyl Acetate and Reynold's Lead Citrate. Images were acquired using an Ultrascan 1000, 2K x 2K CCD digital camera, on a JEOL JEM1400 TEM operated at 80kV. Micrographs of 140-600 cells per strain were analyzed and representative images were selected for publication. Particle diameters were measured single-blind using FIJI version 2.3.0 (17) by counting at least 60 particles from all cells visible in the field of view (1-3 cells) in at least two separate micrographs.

FIG. S1

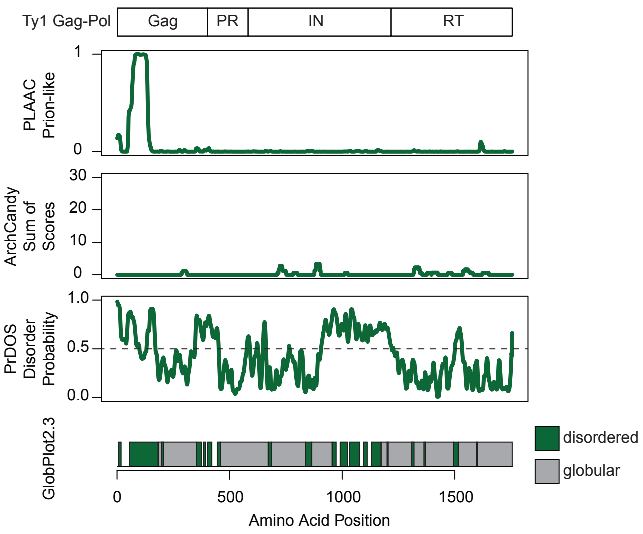

Fig S1. PrLD predictions for Ty1. Schematic of the Ty1 Gag-Pol p199 polyprotein (*top*). Below are bioinformatic analyses aligned with the schematic above: yeast prion-like amino acid composition (PLAAC), predicted amyloidogenic regions (ArchCandy), predicted protein disorder (PrDOS), predicted disordered (green) and globular (grey) regions (GlobPlot2.3).

FIG. S2

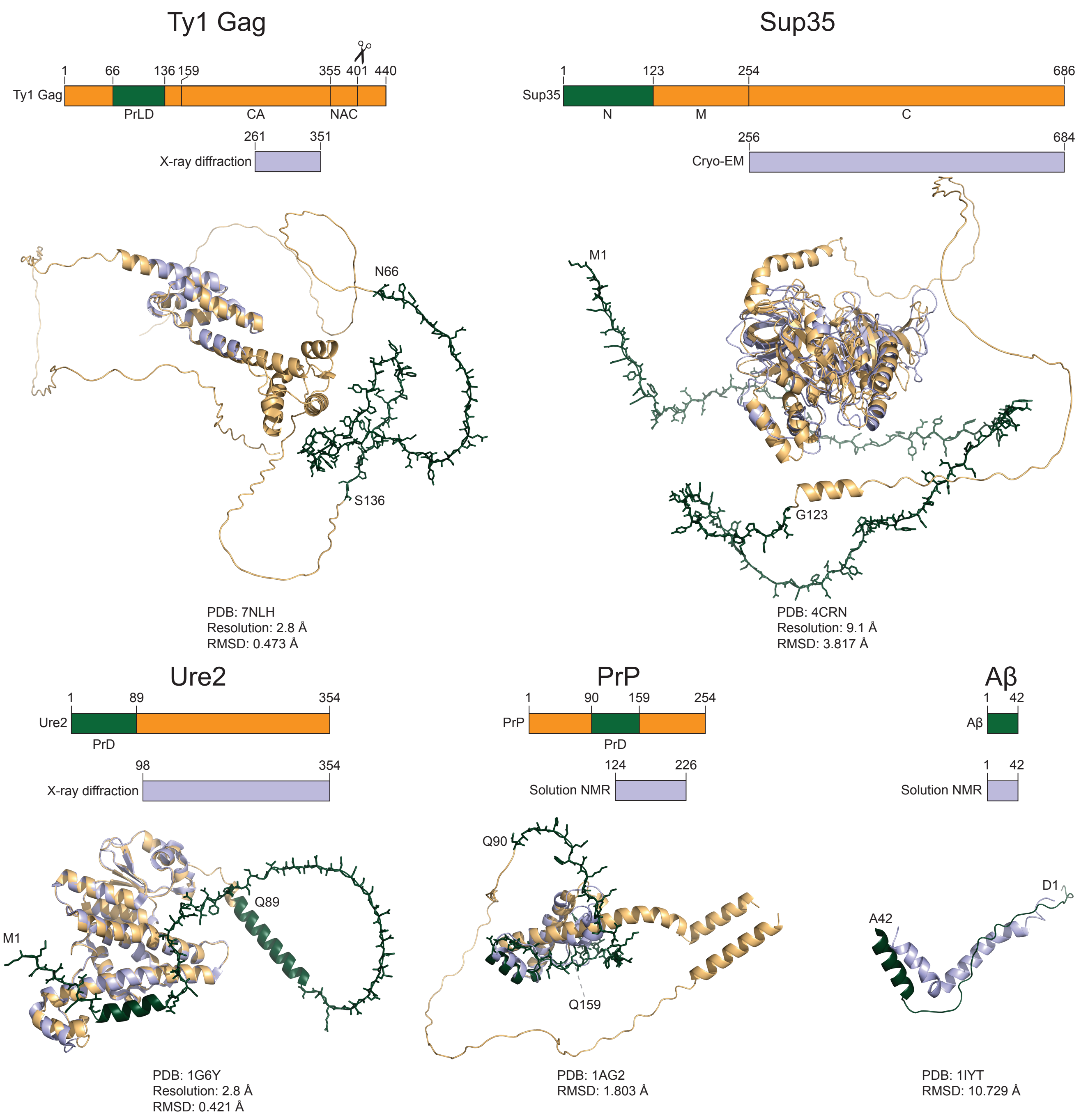

Fig S2. Prionogenic domains are intrinsically disordered in experimental and predicted protein structures. Schematics of protein domains (*top*) and experimentally determined protein structures with the methodology are noted (*bottom*). Amino acid coordinates are shown above cartoon representations of structures predicted by AlphaFold (orange) aligned to published structures (blue). Prion domains are colored in dark green, and their predicted disordered loops are shown in stick representation to aid visualization. PDB accession numbers and reported resolutions for published structures, and RMSD over the common residues between the published and predicted structures, are indicated.

FIG. S3

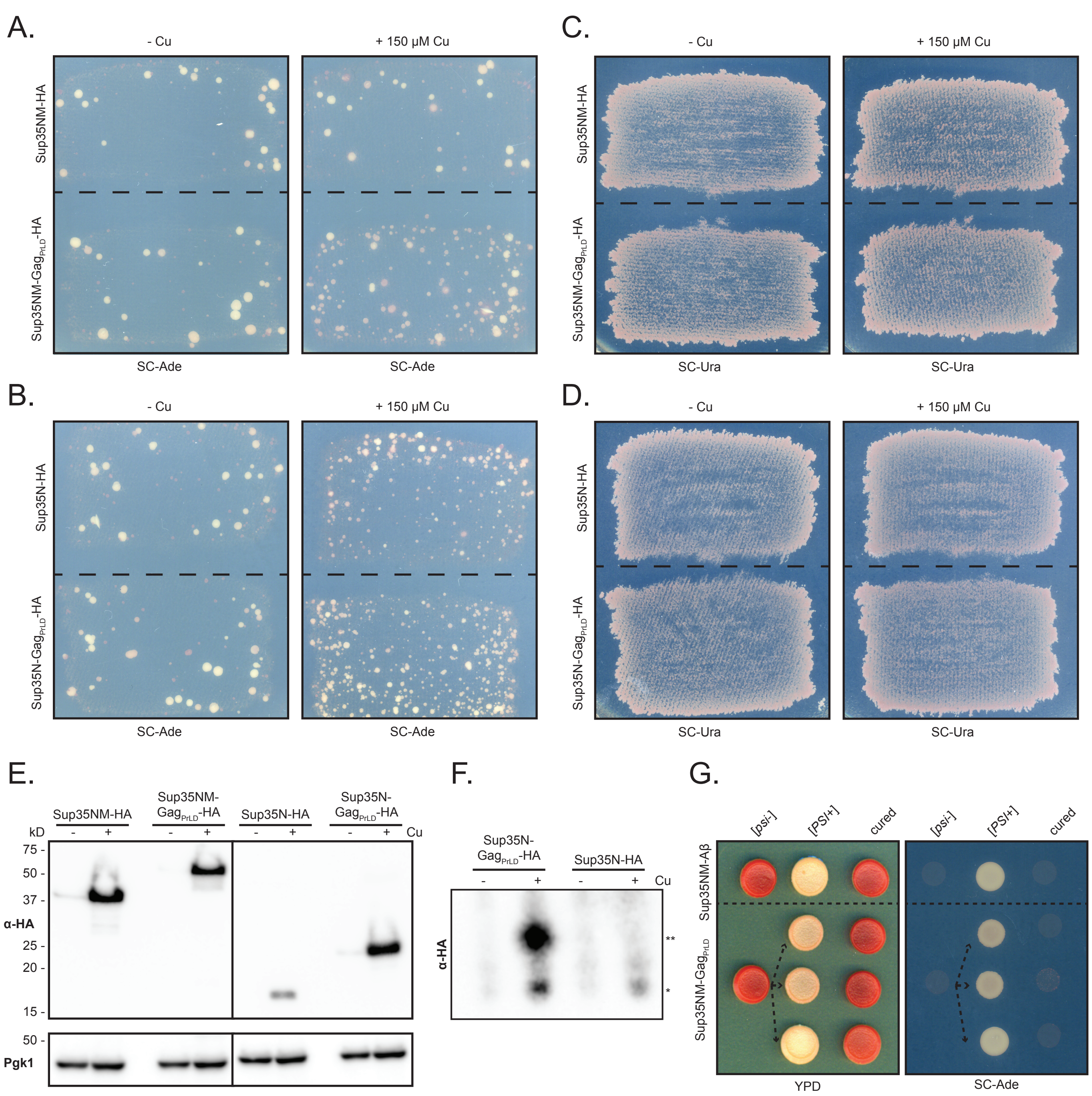

Fig S3. Gag_{PrLD} nucleates a Sup35-based prion reporter. (*A-D*) Qualitative prionogenesis of Sup35 fusions; growth on SC-Ade indicates either a suppressor mutation or $[PSI^+]$ prionogenesis. Expression of Sup35 fusions were induced with 150 µM $CuSO_4$. SC-Ura plates demonstrate similar number of cells printed. A representative image of at least 3 experiments is shown. (*E*) Protein extracts prepared from cells grown with or without 100 µM $CuSO_4$ were immunoblotted for the HA tag. Pgk1 serves as a loading control. Migration of molecular weight standards is shown alongside the immunoblots. A representative image of at least 3 replicates is shown. (*F*) SDD-AGE analysis of Sup35N-HA with and without Gag_{PrLD} fusion. Expression of Sup35 fusions were induced with 100 µM $CuSO_4$. Monomers (*) and high-molecular weight aggregates (**) of chimeric proteins were detected with anti-HA antibody. A representative image of at least 3 experiments is shown. (*D*) Curing of Ade+ colonies by guanidine hydrochloride (GdHCl) of Sup35NM chimeras. One $[psi^-]$ Sup35NM-Aβ fusion control strain is shown induced to $[PSI^+]$ and cured. Three independent inductions of a $[psi^-]$ Sup35NM-GagPrLD fusion are shown induced to $[PSI^+]$ and cured. $[PSI^+]$ yeast cells are white on YPD and grow on SC-Ade while $[psi^-]$ and cured cells are red on YPD and do not grow on SC-Ade.

FIG. S4

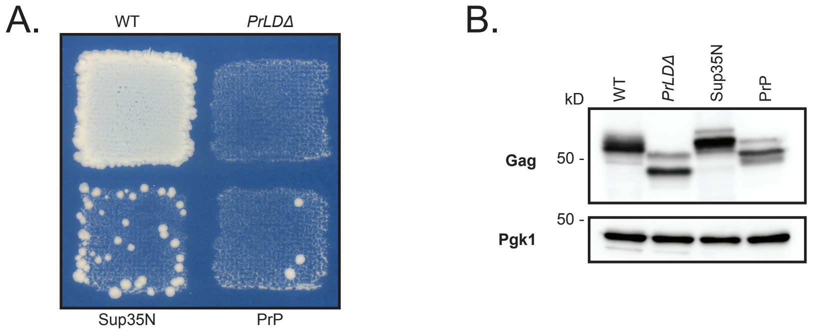

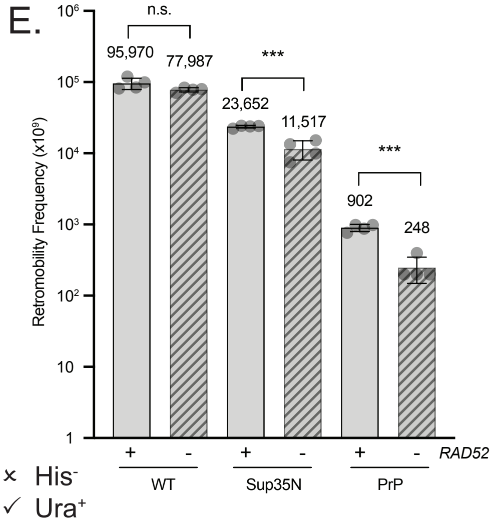

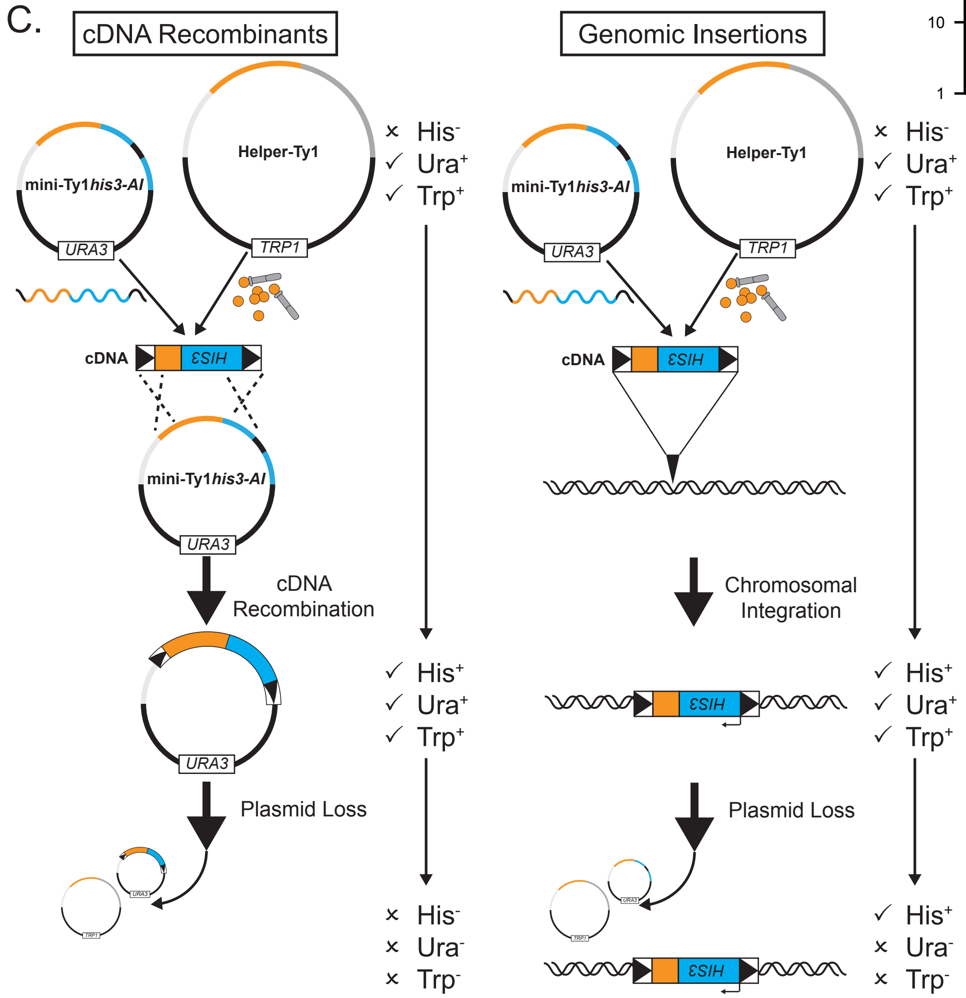

D.

Strain	WT	Sup35N	PrP
cDNA Reombinants	4	14	12
Genomic Insertions	96	86	88
Total Events	100	100	100
p-value		0.024	0.065

Fig S4. Gag chimeras likely disrupt Ty1 RNA functions and increase cDNA recombination with plasmid-borne mini-Ty1*his3-AI*. (*A*) Qualitative retromobility of chimeric Gag constructs in a single pGTy1*his3-AI* plasmid. Growth on media lacking histidine indicates a retromobility event. A representative image of at least 3 replicates is shown. (*B*) Protein extracts prepared from galactose-induced yeast cells expressing the indicated Gag constructs in a single plasmid were immunoblotted for Gag. Pgk1 serves as a loading control. Migration of molecular weight standards is shown alongside the immunoblots. A representative image of at least 3 replicates is shown. (*C*) Schematic of two major retromobility pathways that lead to His^+ cells detected in retromobility assays using Ty-less strains. cDNA recombination and genomic insertion can be differentiated by allowing for plasmid loss after a retromobility event and testing for the retention of growth on medium lacking histidine. (*D*) Table indicating the ratio of cDNA recombinants versus genomic insertions, *p*-values are compared to wildtype. (*E*) Quantitative mobility assay of galactose-induced cells. Each bar represents the mean of four independent measurements, displayed as points, and the error bar ± the standard deviation. Adjusted retromobility frequency is indicated above the bars. Significance is calculated from a two-sided Student's *t*-test (n.s. not significant, ****p* < 0.001. Exact *p*-values are provided in Supplementary Table 1).

FIG. S5

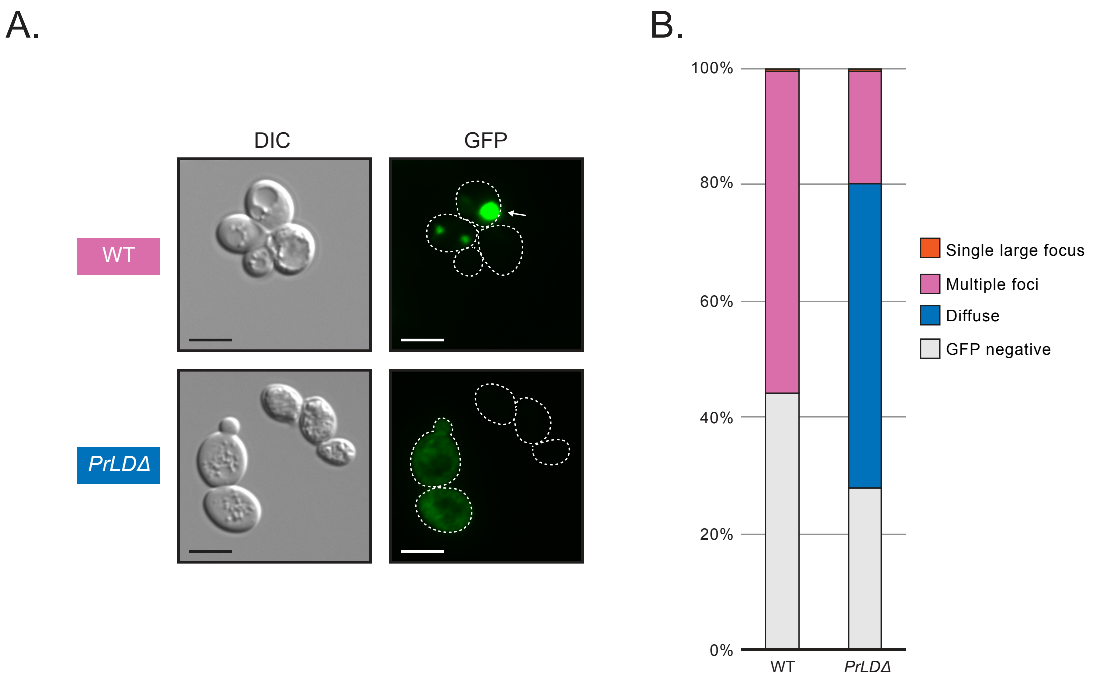

Fig S5. Chimeric Gag-GFP after 48 hr galactose induction. (*A*) Live-cell yeast fluorescence microscopy of strains expressing chimeric Gag-GFP after 48 hr galactose induction. Normaski (DIC) and GFP channels are shown with cell outlines added to GFP channels based on DIC images. The strain labels are colored to match the most common foci observed. White arrows indicate cells with a single large focus. Scale bars represent 5 µm. (*B*) Quantitation of categories of foci observed as a percentage. The multiple foci category includes cells with multiple large foci, one or more small foci, or a combination of both sizes. Exact cell counts are provided in Supplementary Table 2.

FIG. S6

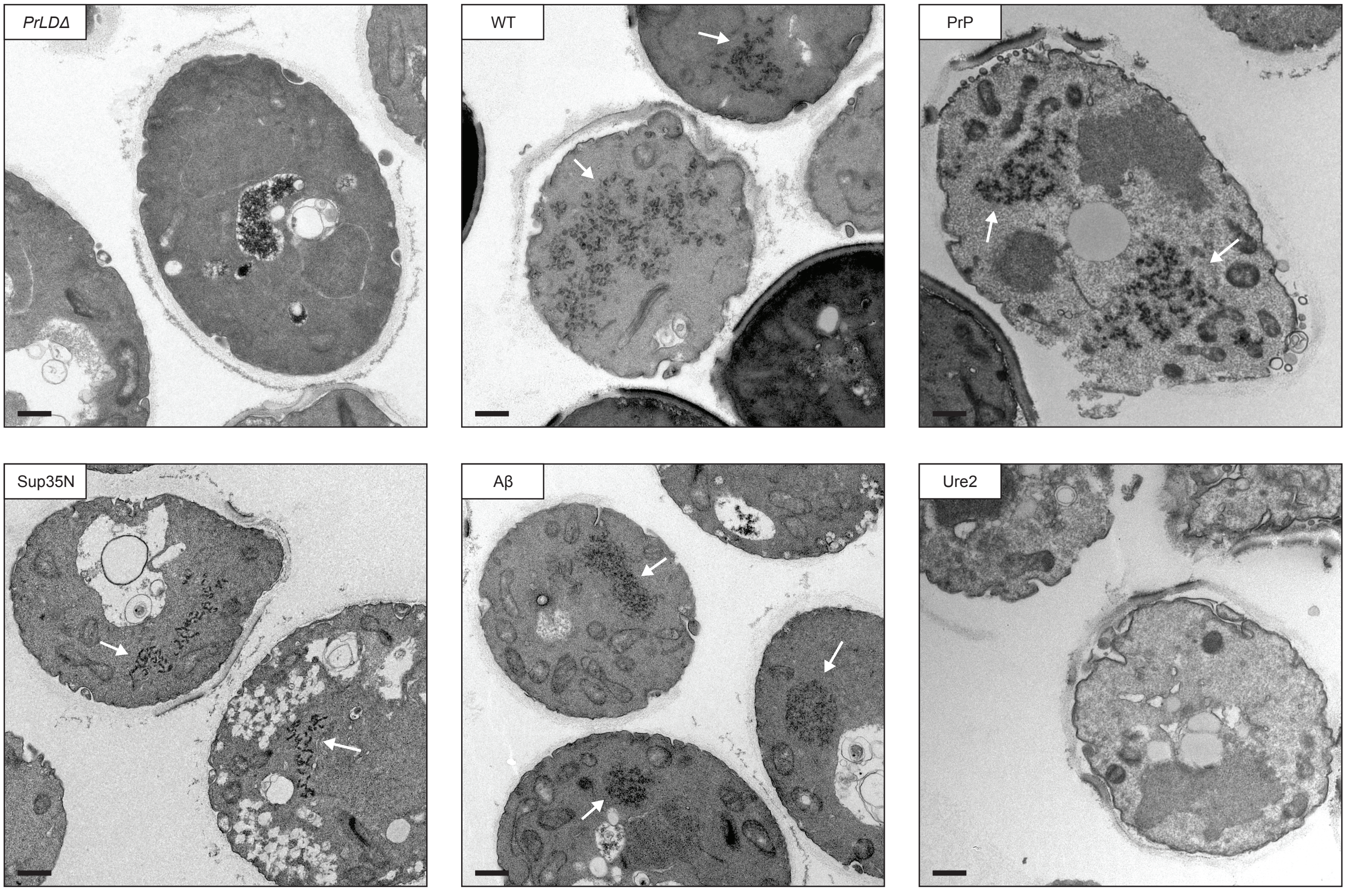

Fig S6. Thin-section TEM of Gag-GFP strains. Thin-section TEM of 24 hr galactose-induced cells expressing Gag-GFP chimeras. Representative cells are shown. White arrows indicate Gag-GFP structures in the cells. Scale bars represent 500 nm.

FIG. S7

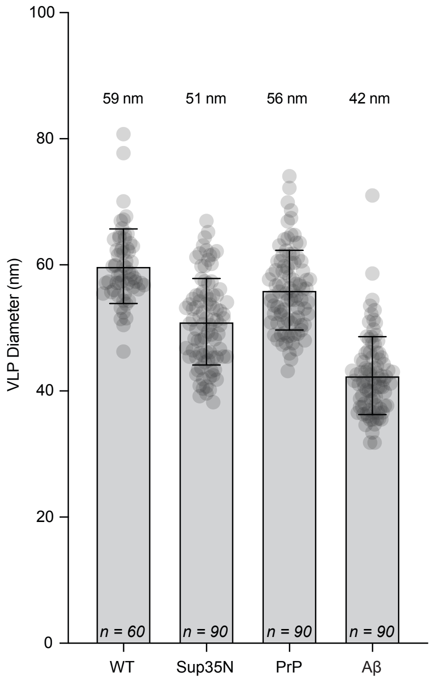

Fig S7. VLP diameter of Gag-PrLD chimeras. Diameter measurements of particles in galactose-induced cells expressing Gag chimeras visualized by thin-section TEM. Each bar represents the mean diameter, displayed as points, and the error bar ± the standard deviation. The median diameter is noted above each bar, the number of particles measured is noted at the base of each bar. Particles from all strains are significantly smaller as calculated from a two-sided Student's *t*-test compared with WT.

Supplementary Table S1. Retromobility frequencies.

Strain	Label	Retromobility Frequency	Std Dev	*p*-value[a]	Biological replicates
DG4457	WT	6.46×10^{-5}	2.72×10^{-5}	Reference	20
DG4197	*PrLDΔ*	8.75×10^{-9}	2.47×10^{-8}	4.75×10^{-7}	8
DG4198	Sup35N	6.57×10^{-5}	3.59×10^{-5}	0.933	8
DG4201	Ure2	0	0	4.74×10^{-7}	8
DG4242	PrP	1.22×10^{-6}	5.59×10^{-7}	6.51×10^{-7}	8
DG4241	Aβ	0	0	4.74×10^{-7}	8
SLBY460	WT/*RAD52*$^{+}$	9.60×10^{-5}	1.75×10^{-5}	Reference[b]	4
SLBY463	WT/*rad52*$^{-}$	7.80×10^{-5}	5.31×10^{-6}	0.097	4
SLBY461	Sup35N/*RAD52*$^{+}$	2.37×10^{-5}	9.75×10^{-7}	Reference	4
SLBY464	Sup35N/*rad52*$^{-}$	1.15×10^{-5}	3.46×10^{-6}	5.16×10^{-4}	4
SLBY462	PrP/*RAD52*$^{+}$	9.02×10^{-7}	1.05×10^{-7}	Reference	4
SLBY465	PrP/*rad52*$^{-}$	2.48×10^{-7}	9.93×10^{-8}	1.01×10^{-4}	4

[a] Calculated by two-sided Student's *t*-test

[b] *p*-statistics calculated relative to corresponding *RAD52*$^{+}$ strain

Supplementary Table S2. Gag-GFP chimera fluorescent microscopy cell counts.

Strain	Label	GFP negative	Diffuse	Multiple foci[a]	Single large focus	Total cells
24 hr induction						
DG4513	WT	32	0	307	2	341
DG4514	*PrLDΔ*	48	293	50	0	391
DG4515	Sup35N	24	4	276	5	309
DG4516	Ure2	38	320	7	0	365
DG4517	PrP	44	45	196	48	333
DG4518	Aβ	65	23	127	89	304
48 hr induction						
DG4513	WT	108	0	135	1	244
DG4514	*PrLDΔ*	83	159	58	1	301

[a] This category includes multiple large foci, one or more small foci, or a combination of both sizes.

Supplementary Table S3. Yeast strains used in this study.

Strain	Genotype	Plasmids	Source
GT409	*Saccharomyces cerevisiae MAT***a** *ade1-14 his3 leu2-3,112 lys2 trp1 ura3-52* [*psi*$^-$ *pin*$^-$]		(6)
SLBY294	GT409	SLBB027	This study
SLBY286	GT409	SLBB021	This study
SLBY295	GT409	SLBB028	This study
SLBY287	GT409	SLBB022	This study
SLBY285	GT409	SLBB020	This study
DG4218	GT409	BDG1691	This study
DG4219	GT409	BDG1701	This study
DG3582	*Saccharomyces paradoxus MAT*α *gal3 his3-Δ200hisG trp1-1* ura3* Ty-less		(8)
DG4457	DG3582	BDG1647, BDG1781	This study
DG4197	DG3582	BDG1647, BDG1680	This study
DG4198	DG3582	BDG1647, BDG1681	This study
DG4201	DG3582	BDG1647, BDG1684	This study
DG4241	DG3582	BDG1647, BDG1712	This study
DG4242	DG3582	BDG1647, BDG1713	This study
DG4441	DG3582	BDG673, BDG674	This study
DG4156	DG3582	BDG598	This study
DG4447	DG3582	SLBB050	This study
DG4448	DG3582	SLBB051	This study
DG4449	DG3582	SLBB052	This study
DG4513	DG3582	BDG1799	This study
DG4514	DG3582	BDG1800	This study
DG4515	DG3582	BDG1801	This study
DG4516	DG3582	BDG1802	This study
DG4517	DG3582	BDG1803	This study
DG4518	DG3582	BDG1804	This study
DG2204	*Saccharomyces paradoxus MAT***a** *gal3 his3-Δ200hisG trp1-1* ura3* Ty-less		This study
SLBY460	DG2204	BDG1647, BDG1781	This study
SLBY461	DG2204	BDG1647, BDG1681	This study
SLBY462	DG2204	BDG1647, BDG1713	This study
DG2689	DG2204 *rad52::KanMX*		This study
SLBY463	DG2689	BDG1647, BDG1781	This study
SLBY464	DG2689	BDG1647, BDG1681	This study
SLBY465	DG2689	BDG1647, BDG1713	This study

Supplementary Table S4. Plasmids used in this study.

**Plasmid**	**Description**	**Markers**	**Source**
pBDG598	pGTy1*his3-AI*	*URA3/2μ*	(12)
pSLBB050	pBDG598-*PrLDΔ*	*URA3/2μ*	This study
pSLBB051	pBDG598-Sup35N_{2-123}	*URA3/2μ*	This study
pSLBB052	pBDG598-PrP_{90-159}	*URA3/2μ*	This study
pBDG1647	pGTy1*hisAI*-Δnt818-5463	*URA3/2μ*	(10)
pBDG1781	pGTy1nt.241-5561	*TRP1/2μ*	This study
pBDG1680	pBDG1781-*PrLDΔ*	*TRP1/2μ*	This study
pBDG1681	pBDG1781-Sup35N_{2-123}	*TRP1/2μ*	This study
pBDG1684	pBDG1781-$Ure2_{17-76}$	*TRP1/2μ*	This study
pBDG1712	pBDG1781-$A\beta_{1-42}$	*TRP1/2μ*	This study
pBDG1713	pBDG1781-PrP_{90-159}	*TRP1/2μ*	This study
pBDG673	pRS424	*TRP1/2μ*	(11)
pBDG674	pRS426	*URA3/2μ*	(11)
pBDG1691	pCUP1-SUP35NM-$A\beta_{1-42}$	*URA3/CEN*	(6)
pBDG1701	pCUP1-SUP35NM-Gag_{PrLD}	*URA3/CEN*	This study
pSLBB020	pCUP1-SUP35NM-$A\beta_{1-42}$-HA	*URA3/CEN*	This study
pSLBB021	pCUP1-SUP35NM-Gag_{PrLD}-HA	*URA3/CEN*	This study
pSLBB022	pCUP1-SUP35N-Gag_{PrLD}-HA	*URA3/CEN*	This study
pSLBB027	pCUP1-SUP35NM-HA	*URA3/CEN*	[a]
pSLBB028	pCUP1-SUP35N-HA	*URA3/CEN*	(6)
pBDG1799	pGAL-Gag_{1-401}-GFP	*HIS3/CEN*	This study
pBDG1800	pBDG1799-*PrLDΔ*	*HIS3/CEN*	This study
pBDG1801	pBDG1799-Sup35N_{2-123}	*HIS3/CEN*	This study
pBDG1802	pBDG1799-$Ure2_{17-76}$	*HIS3/CEN*	This study
pBDG1803	pBDG1799-PrP_{90-159}	*HIS3/CEN*	This study
pBDG1804	pBDG1799-$A\beta_{1-42}$	*HIS3/CEN*	This study

[a] Kindly provided by Y. Chernoff.

Supplementary Table S5. Primers used in this study.

Construct	Description	Oligos (5' - 3')
SLBP0045	SupM-PrLDF	GAAGTGGATGACGAAGTTGAATTCAACCCCCATCATGCCTCTCC
SLBP0046	SupN-PrLDF	CAACCACAGTCTCAAGGTGAATTCAACCCCCATCATGCCTCTCC
SLBP0047	Sup-PrLDR	CACCGCGGTGGCGGCCGCTCTAGATTATGATGATGGATACTGCGG
SLBP0086	HAtag-SacII	TACCCATACGACGTACCAGATTACGCTTGACCGCGGTGGAGCTCCAA
SLBP0087	GagPrLD-HA	CAGTATCCATCATCATACCCATACGACGTA
SLBP0088	AB42-HA	GGTGTTGTCATAGCGTACCCATACGACGTA
SLBP0194	Ty1 779 Rev	CATATCAGAGTCCGCTGAGG
SLBP0116	Ty1 835 Rev	GGAAAGTCATTAGGTGAGG
SLBP0117	GTy1 Xho Fwd	GTATTACTTCTTATTCCTCGAGG
SLBP0221	pRS Fwd	TTGGGTACCGGGCCC
SLBP0222	pRS Rev	AAAGCTGGAGCTCCACC

Supplementary Table S6. Gene fragments used in this study.

Construct	Description	Oligos (5' - 3')
SLBG0024	Ty1 XhoI PrLD	ACTTCTTATTCCTCTACCGCCTCGAGGAGAACTTCTAGTATATTCTGTATACCT<br>AATATTATAGCCTTTATCAACAATGGAATCCCAACAATTATCTCAACATTCACC<br>CAATTCTCATGGTAGCGCCTGTGCTTCGGTTACTTCTAAGGAAGTCCACACAAA<br>TCAAGATCCGTTAGACGTTTCAGCTTCCAAAACAGAAGAATGTGAGAAGGCTTC<br>CACTAAGGCTAACTCTCAACAGACAACAACACCTGCTTCATCAGCTGTTCCAGA<br>G
SLBG0025	Ty1 702-840	GTTGGAACGCCTCTGAGCACTCCATCACCTGAGTCAGGTAATACATTTACTGAT<br>TCATCCTCAGCGGACTCTGATATGACATCCACTAAAAAATATGTCAGACCACCA<br>CCAATGTTAACCTCACCTAATGACTTTCCAA
SLBG0026	Ty1 Sup35N	ACAACACCTGCTTCATCAGCTGTTCCAGAGTCGGATTCAAACCAAGGCAACAAT<br>CAGCAAAACTACCAGCAATACAGCCAGAACGGTAACCAACAACAAGGTAACAAC<br>AGATACCAAGGTTATCAAGCTTACAATGCTCAAGCCCAACCTGCAGGTGGGTAC<br>TACCAAAATTACCAAGGTTATTCTGGGTACCAACAAGGTGGCTATCAACAGTAC<br>AATCCCGACGCCGGTTACCAGCAACAGTATAATCCTCAAGGAGGCTATCAACAG<br>TACAATCCTCAAGGCGGTTATCAGCAGCAATTCAATCCACAAGGTGGCCGTGGA<br>AATTACAAAAACTTCAACTACAATAACAATTTGCAAGGATATCAAGCTGGTTTC<br>CAACCACAGTCTCAAGGTGTTGGAACGCCTCTGAGCACTCCATCACCT
SLBG0029	Ty1 Ure2	ACAACACCTGCTTCATCAGCTGTTCCAGAGCGTCAAGTAAACATAGGAAACAGG<br>AACAGTAATACAACCACCGATCAAAGTAATATAAATTTTGAATTTTCAACAGGT<br>GTAAATAATAATAATAATAACAATAGCAGTAGTAATAACAATAATGTTCAAAAC<br>AATAACAGCGGCCGCAATGGTAGCCAAAATAATGATAACGAGAATAATGTTGGA<br>ACGCCTCTGAGCACTCCATCACCT
SLBG0030	Ty1 PrLDΔ	ACTTCTTATTCCTCTACCGCCTCGAGGAGAACTTCTAGTATATTCTGTATACCT<br>AATATTATAGCCTTTATCAACAATGGAATCCCAACAATTATCTCAACATTCACC<br>CAATTCTCATGGTAGCGCCTGTGCTTCGGTTACTTCTAAGGAAGTCCACACAAA<br>TCAAGATCCGTTAGACGTTTCAGCTTCCAAAACAGAAGAATGTGAGAAGGCTTC<br>CACTAAGGCTAACTCTCAACAGACAACAACACCTGCTTCATCAGCTGTTCCAGA<br>GGTTGGAACGCCTCTGAGCACTCCATCACCTGAGTCAGGTAATACATTTACTGA<br>TTCATCCTCAGCGGACTCTGATATGACATCCACTAAAAAATATGTCAGACCACC<br>ACCAATGTTAACCTCACCTAATGACTTTCCAA
SLBG0035	Ty1 Abeta	CTTCATCAGCTGTTCCAGAGGATGCAGAGTTCCGACATGACTCAGGATATGAAG<br>TTCATCATCAAAAATTGGTGTTCTTTGCAGAAGATGTGGGTTCAAACAAAGGTG<br>CAATCATTGGACTCATGGTGGGCGGTGTTGTCATAGCGGTTGGAACGCCTCTGA<br>GCAC
SLBG0036	Ty1 PrP	CTTCATCAGCTGTTCCAGAGCAGGGAGGAGGTACACACAATCAGTGGAATAAAC<br>CAAGCAAACCGAAGACGAACTTGAAACACGTCGCCGGCGCCGCGGCTGCAGGGG<br>CGGTTGTTGGAGGACTTGGTGGGTATATGCTGGGCAGTGCTATGAGCCGTCCCA<br>TGATCCATTTTGGTAACGATTGGGAAGACCGTTATTATAGGGAGAACATGTATA<br>GGTACCCTAATCAGGTTGGAACGCCTCTGAGCAC

SI References

1. A. K. Lancaster, A. Nutter-Upham, S. Lindquist, O. D. King, PLAAC: a web and command-line application to identify proteins with prion-like amino acid composition. *Bioinformatics* **30**, 2501–2 (2014).
2. A. B. Ahmed, N. Znassi, M. T. Château, A. v. Kajava, A structure-based approach to predict predisposition to amyloidosis. *Alzheimer's and Dementia* **11**, 681–690 (2015).
3. T. Ishida, K. Kinoshita, PrDOS: prediction of disordered protein regions from amino acid sequence. *Nucleic Acids Res* **35**, W460-4 (2007).
4. R. Linding, R. B. Russell, V. Neduva, T. J. Gibson, GlobPlot: Exploring protein sequences for globularity and disorder. *Nucleic Acids Res* **31**, 3701–8 (2003).
5. C. Guthrie, G. Fink, Guide to yeast genetics and molecular biology. *Methods Enzymol* **194**, 1–863 (1991).
6. P. Chandramowlishwaran, *et al.*, Mammalian amyloidogenic proteins promote prion nucleation in yeast. *J Biol Chem* **293**, 3436–3450 (2018).
7. J. Chen, *et al.*, Genome Assembly of the Ty1-Less Saccharomyces paradoxus Strain DG1768. *Microbiol Resour Announc* **11**, e0086821 (2022).
8. A. Saha, *et al.*, A trans-dominant form of Gag restricts Ty1 retrotransposition and mediates copy number control. *J Virol* **89**, 3922–38 (2015).
9. D. J. Garfinkel, K. Nyswaner, J. Wang, J.-Y. Cho, Post-transcriptional cosuppression of Ty1 retrotransposition. *Genetics* **165**, 83–99 (2003).
10. J. Gumna, K. J. Purzycka, H. W. Ahn, D. J. Garfinkel, K. Pachulska-Wieczorek, Retroviral-like determinants and functions required for dimerization of Ty1 retrotransposon RNA. *RNA Biol*, 1–15 (2019).
11. C. B. Brachmann, *et al.*, Designer deletion strains derived from Saccharomyces cerevisiae S288C: a useful set of strains and plasmids for PCR-mediated gene disruption and other applications. *Yeast* **14**, 115–32 (1998).
12. M. J. Curcio, D. J. Garfinkel, Single-step selection for Ty1 element retrotransposition. *Proc Natl Acad Sci U S A* **88**, 936–40 (1991).
13. R. Halfmann, S. Lindquist, Screening for amyloid aggregation by Semi-Denaturing Detergent-Agarose Gel Electrophoresis. *J Vis Exp*, 11–13 (2008).
14. F. Faul, E. Erdfelder, A. Buchner, A.-G. Lang, Statistical power analyses using G*Power 3.1: tests for correlation and regression analyses. *Behav Res Methods* **41**, 1149–60 (2009).
15. A. Ohashi, J. Gibson, I. Gregor, G. Schatz, Import of proteins into mitochondria. The precursor of cytochrome c1 is processed in two steps, one of them heme-dependent. *J Biol Chem* **257**, 13042–7 (1982).
16. P. Bastin, A. Bagherzadeh, K. R. Matthews, K. Gull, A novel epitope tag system to study protein targeting and organelle biogenesis in Trypanosoma brucei. *Mol Biochem Parasitol* **77**, 235–239 (1996).
17. J. Schindelin, *et al.*, Fiji: an open-source platform for biological-image analysis. *Nat Methods* **9**, 676–82 (2012).
